# Discrete Ricci curvatures capture age-related changes in human brain functional connectivity networks

**DOI:** 10.3389/fnagi.2023.1120846

**Published:** 2023-05-24

**Authors:** Yasharth Yadav, Pavithra Elumalai, Nitin Williams, Jürgen Jost, Areejit Samal

**Affiliations:** ^1^The Institute of Mathematical Sciences (IMSc), Chennai, India; ^2^Indian Institute of Science Education and Research (IISER), Pune, India; ^3^Department of Computer Science, Helsinki Institute of Information Technology, Aalto University, Espoo, Finland; ^4^Department of Neuroscience and Biomedical Engineering, Aalto University, Espoo, Finland; ^5^Max Planck Institute for Mathematics in the Sciences, Leipzig, Germany; ^6^The Santa Fe Institute, Santa Fe, NM, United States; ^7^Homi Bhabha National Institute (HBNI), Mumbai, India

**Keywords:** Forman-Ricci curvature, Ollivier-Ricci curvature, healthy aging, resting-state fMRI, functional connectivity networks, MPI-LEMON, non-invasive brain stimulation, motor performance

## Abstract

**Introduction:**

Geometry-inspired notions of discrete Ricci curvature have been successfully used as markers of disrupted brain connectivity in neuropsychiatric disorders, but their ability to characterize age-related changes in functional connectivity is unexplored.

**Methods:**

We apply Forman-Ricci curvature and Ollivier-Ricci curvature to compare functional connectivity networks of healthy young and older subjects from the Max Planck Institute Leipzig Study for Mind-Body-Emotion Interactions (MPI-LEMON) dataset (*N* = 225).

**Results:**

We found that both Forman-Ricci curvature and Ollivier-Ricci curvature can capture whole-brain and region-level age-related differences in functional connectivity. Meta-analysis decoding demonstrated that those brain regions with age-related curvature differences were associated with cognitive domains known to manifest age-related changes—movement, affective processing, and somatosensory processing. Moreover, the curvature values of some brain regions showing age-related differences exhibited correlations with behavioral scores of affective processing. Finally, we found an overlap between brain regions showing age-related curvature differences and those brain regions whose non-invasive stimulation resulted in improved movement performance in older adults.

**Discussion:**

Our results suggest that both Forman-Ricci curvature and Ollivier-Ricci curvature correctly identify brain regions that are known to be functionally or clinically relevant. Our results add to a growing body of evidence demonstrating the sensitivity of discrete Ricci curvature measures to changes in the organization of functional connectivity networks, both in health and disease.

## 1. Introduction

The proportion of older adults or elderly is increasing across the world. Globally, the number of adults aged 65 years or above surpassed the number of children under the age of 5 for the first time in 2018. Further, the share of the global population aged 65 years or above is projected to rise from 10% in 2022 to 16% in 2050 (Ritchie and Roser, 2019; United Nations Department of Economic and Social Affairs, Population Division, 2022). This upward shift in the age distribution of the global population makes it crucial (Cabeza et al., 2016) to identify the neuronal correlates of age-related decline in cognition, perception and motor performance. The advent of cutting-edge neuroimaging technologies has facilitated the study of anatomical and functional changes in the human brain during the healthy aging process (Grady, 2012; Tromp et al., 2015). Specifically, studies employing functional magnetic resonance imaging (fMRI) methods (Spreng et al., 2010; Eyler et al., 2011; Rodriguez-Sabate et al., 2022) have shed light on the altered activity of brain regions that are necessary for optimal cognitive performance in elderly individuals, including the prefrontal, medial temporal and parietal cortices (Nyberg et al., 2010; Grady, 2012; Addis et al., 2015). There is also an increasing interest in the development of novel intervention strategies for reversing the effects of age-related functional decline. Non-invasive brain stimulation techniques such as transcranial direct current stimulation (tDCS), transcranial alternating current stimulation (tACS), and transcranial magnetic stimulation (TMS) offer an attractive option to modulate neuronal plasticity and improve learning processes in older individuals (Nitsche et al., 2008; Reis et al., 2009; Zimerman and Hummel, 2010). Neuroimaging techniques such as fMRI can not only record the activity of individual regions in the human brain, but also capture the correlations between the activities of these brain regions (Logothetis, 2008), thereby revealing the underlying functional connectivity networks (FCNs). Numerous efforts have been undertaken to characterize age-related changes in FCNs using concepts and tools from network science and graph theory (Bassett and Sporns, 2017). Remarkably, graph-theoretic studies on resting-state fMRI (rs-fMRI) datasets of healthy young and healthy older individuals have demonstrated age-related changes in several network-based measures including clustering coefficient, shortest path length, global efficiency, local efficiency, and modularity (Achard and Bullmore, 2007; Sala-Llonch et al., 2014; Song et al., 2014; Geerligs et al., 2015; Li et al., 2016). Recent graph-theoretic studies have combined data from diffusion tensor imaging (DTI) and rs-fMRI scans of healthy aging participants in the Max Planck Institute Leipzig Study for Mind-Body-Emotion Interactions (MPI-LEMON) (Babayan et al., 2019) to analyze the influence of white matter hyperintensities (WMH) and global fractional anisotropy (gFA) on resting-state FCNs (Porcu et al., 2020, 2021).

In recent years, several geometric notions of *discrete Ricci curvature* (Forman, 2003; Ollivier, 2007; Sreejith et al., 2016; Samal et al., 2018) have been introduced as novel tools for the analysis of complex networks (Bianconi and Rahmede, 2017; Boguñá et al., 2021). Discrete Ricci curvatures allow for a rigorous characterization of network structure by accounting for higher-order correlations in a network (Kartun-Giles and Bianconi, 2019), and have found important applications such as community detection in networks (Ni et al., 2019; Sia et al., 2019; Tian et al., 2022) and indicators of critical events in financial markets (Sandhu et al., 2016; Samal et al., 2021). Forman-Ricci curvature (FRC) (Forman, 2003; Sreejith et al., 2016) and Ollivier-Ricci curvature (ORC) (Ollivier, 2007) are the two commonly-used notions of discrete Ricci curvature for analysis of complex real-world networks. Multiple studies have utilized FRC and ORC to measure changes in structural and functional brain connectivity during neurodevelopmental disorders (Farooq et al., 2019, 2020; Simhal et al., 2020, 2022; Chatterjee et al., 2021; Elumalai et al., 2022). For example, Chatterjee et al. (2021) revealed differences in FRC in the FCNs of individuals with attention deficit hyperactivity disorder (ADHD) compared to the FCNs of healthy controls. Recently, Elumalai et al. (2022) demonstrated that FRC can capture differences in FCNs of individuals with autism spectrum disorder (ASD) compared to FCNs of typically developing controls, and could be used to identify brain regions clinically relevant to ASD. Additionally, Simhal et al. (2022) used ORC to characterize changes in white matter connectivity after intravenous cord blood infusion in children with ASD. FRC and ORC have also been applied to brain networks of healthy populations. Specifically, Lohmann et al. (2021) applied FRC to predict the intelligence of healthy human subjects using fMRI data, and Farooq et al. (2019) applied ORC to identify changes in brain structural connectivity during healthy aging. However, no previous study has assessed the ability of discrete Ricci curvatures to characterize FCN alterations during healthy aging.

**Figure 1:**
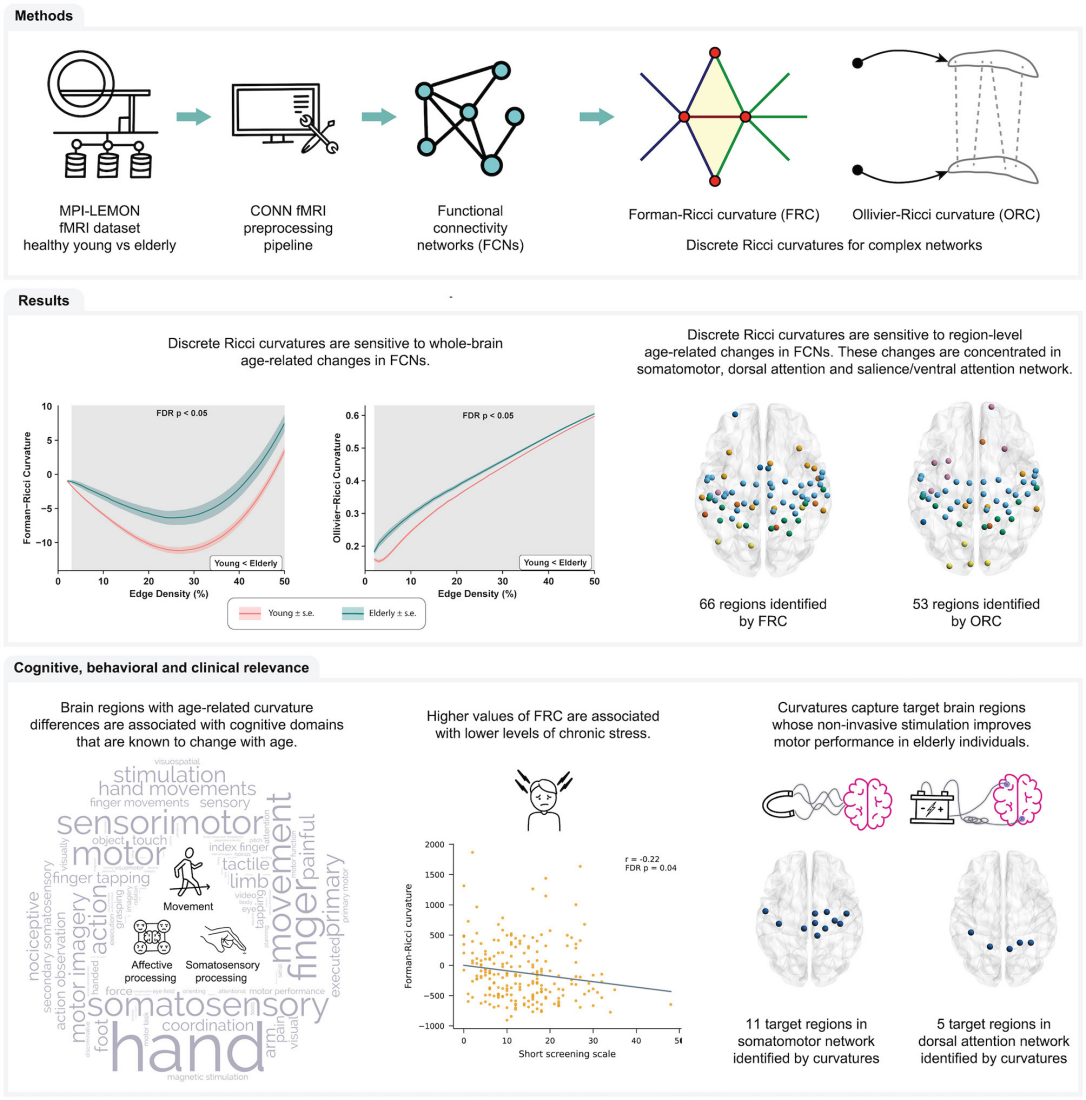
Summary of workflow and main results obtained in this study. Raw rs-fMRI scans of young participants and elderly participants were obtained from the MPI-LEMON dataset and preprocessed using the CONN toolbox. The preprocessed rs-fMRI scan of each participant was used to construct resting state functional connectivity networks (FCNs) at varying edge densities. Next, we computed and compared Forman-Ricci curvature (FRC) and Ollivier-Ricci curvature (ORC) across the FCNs of the young and elderly groups. We found that both FRC and ORC show whole-brain and region-level differences in the FCNs of young and elderly participants. Additionally, we found that age-related differences in FRC and ORC are associated with the cognitive domains of movement, affective processing and somatosensory processing. Notably, node FRC shows a significant negative correlation with behavioral test scores of chronic stress. Finally, we showed that FRC and ORC can capture brain regions whose non-invasive stimulation is known to improve motor performance of older adults.

In the present work, we apply FRC and ORC to compare resting-state FCNs of healthy young and healthy elderly individuals. We constructed resting-state FCNs by applying a standard fMRI preprocessing pipeline (Elumalai et al., 2022) to raw rs-fMRI images of 225 healthy participants in the MPI-LEMON dataset (Babayan et al., 2019). We estimated whole-brain and region-level FRC and ORC differences between the young and elderly age groups. Next, we used meta-analysis decoding (Yarkoni et al., 2011; Williams et al., 2021) to determine whether discrete Ricci curvatures can identify the cognitive and behavioral domains that are typically associated with healthy aging. Finally, we performed two *post-hoc* analyses based on the results of the meta-analysis decoding. For the first *post-hoc* analysis, we determined whether the node curvatures of brain regions with age-related differences were correlated with performance in behavioral tests of affective processing. Affective processing is known to change with age (Mather, 2012). For the second *post-hoc* analysis, we compared the set of brain regions showing age-related curvature differences, to the set of brain regions whose non-invasive stimulation with tDCS, tACS, or TMS is known to improve motor performance in healthy older adults. Motor performance is also known to change with age (Seidler et al., 2010; Cirillo, 2021). Figure 1 provides a summary of the methods and the main results obtained in this work.

## 2. Materials and methods

We applied Forman-Ricci curvature (FRC) and Ollivier-Ricci curvature (ORC) to measure age-related changes in resting-state functional connectivity networks (FCNs). We constructed the FCNs from raw rs-fMRI images of the subjects acquired from the MPI-LEMON dataset (Babayan et al., 2019). We used the CONN functional connectivity toolbox (Whitfield-Gabrieli and Nieto-Castanon, 2012) to spatially and temporally preprocess the raw rs-fMRI images. Next, we parcellated each preprocessed image into 200 regions of interest (ROIs) or nodes using the Schaefer atlas (Schaefer et al., 2018) and generated a 200 × 200 functional connectivity (FC) matrix for each subject. We constructed the FCNs for each subject using a two-step filtering approach, comprising maximum spanning tree (MST) followed by sparsity-based thresholding. Note that the preprocessing pipeline for raw rs-fMRI images and methodology used to construct the FCNs in this work is identical to our previous work on autism spectrum disorder (Elumalai et al., 2022). In the following, we describe in detail, the procedure we employed to construct and analyze the FCNs.

### 2.1. Participants and imaging dataset

We obtained raw rs-fMRI and anatomical data for 228 participants from the MPI-LEMON dataset (Babayan et al., 2019), comprising 154 young individuals from 20 to 35 years, and 74 elderly individuals from 59 to 77 years. Middle-aged individuals are not included in the MPI-LEMON dataset. We downloaded all the raw rs-fMRI and anatomical data from the “Functional Connectomes Project International Neuroimaging Data-Sharing Initiative/Child Mind Institute” available at the following link: http://fcon_1000.projects.nitrc.org/indi/retro/MPI_LEMON.html. Further, demographic information of the participants such as handedness, formal education, drug test results, smoking status, relationship status, blood sample, blood pressure, and anthropometry is readily available at the following link: https://ftp.gwdg.de/pub/misc/MPI-Leipzig_Mind-Brain-Body-LEMON/.

Next, we excluded the subjects with corrupted or missing raw files in the MPI-LEMON dataset, resulting in 225 subjects (young group: 153 subjects, elderly group: 72 subjects) that we included for subsequent analyses. Note that the participants included in the MPI-LEMON dataset provided written informed consent prior to any data acquisition, including agreement to share their data anonymously.

### 2.2. Raw fMRI data preprocessing

We preprocessed the raw rs-fMRI scans from the MPI-LEMON dataset using the CONN functional connectivity toolbox (Whitfield-Gabrieli and Nieto-Castanon, 2012). The preprocessing pipeline used in this study is identical to our previous work on autism spectrum disorder (Elumalai et al., 2022), and we have published earlier a protocol video explaining this pipeline which is available at: https://youtu.be/ch7-dOA-Vlo.

We spatially preprocessed the raw rs-fMRI scans in four major steps: (i) motion correction, (ii) slice-timing correction, (iii) outlier detection, and (iv) structural and functional segmentation and normalization.

For the motion correction step, we co-registered the raw fMRI scans to the first scan of the first session. We corrected the fMRI scans for motion using six rigid body transformation parameters (three translations and three rotations) that are a part of the SPM12 realign and unwarp procedure (Andersson et al., 2001). For the slice-timing correction step, we corrected temporal misalignment between different slices of the fMRI scans using the SPM12 slice-timing correction procedure (Sladky et al., 2011). For the outlier detection step, we used Artifact Detection Tools (ART)-based outlier detection to mark acquisitions as outliers if they were found to have framewise displacement >0.5 mm or global BOLD signal changes >3 standard deviations. For the segmentation and normalization step, we normalized the images into the Montreal Neurological Institute (MNI) space using the standard procedure (Ashburner and Friston, 2005). Subsequently, we segmented the brain into gray matter, white matter and cerebrospinal fluid (CSF) areas using raw T1-weighted volumes of the anatomical scans and mean BOLD signal of the fMRI scans as reference.

With the CONN toolbox, we extracted the BOLD signals (time series) for each voxel after spatially preprocessing the raw rs-fMRI scans, and performed a temporal preprocessing or denoising step to reduce existing motion effects from BOLD signals. First, we applied an anatomical component-based noise correction procedure (aCompCor), which includes a linear regression step. This step resulted in the removal of five possible noise components (Chai et al., 2012) each from white matter and CSF areas, 12 possible noise components due to estimated subject motion parameters and their first-order derivatives (Friston et al., 1996), and 1 noise component from outlier scans detected earlier (scrubbing) (Power et al., 2014). Second, we removed temporal frequencies <0.008 Hz from the BOLD signals using a high-pass filtering approach.

All the 225 subjects passed the quality assessment checks provided by the CONN toolbox (Ciric et al., 2017; see Supplementary Table 1). Finally, we used the preprocessed fMRI scans of these 225 subjects to construct FC matrices and perform network analysis.

### 2.3. ROI definition and ROI time series estimation

After spatially and temporally preprocessing the raw rs-fMRI scans of the 225 subjects, we partitioned the voxels into cortical parcels so as to define nodes or ROIs with interpretable neurobiological meaning (Luppi and Stamatakis, 2021) and reduce the computational load of further analysis. For this purpose, we used a cortical parcellation atlas provided by Schaefer et al. (2018), which parcellates the brain into 200 distinct ROIs or nodes. Apart from assigning each voxel to one of 200 ROIs, the Schaefer atlas also provides a mapping between each ROI and one of seven resting state networks (RSNs), namely, “default network,” “somatomotor network,” “dorsal attention network,” “salience/ventral attention network,” “visual network,” “limbic network,” and “control network.” With the CONN toolbox, we determined the time series corresponding to each ROI by computing the average time series of its constituent voxels.

### 2.4. Functional connectivity network construction

We constructed a 200 × 200 FC matrix for each of the 225 subjects, by computing the Pearson correlation coefficient between all pairs of ROI time series. The resulting FC matrix can be considered a complete, weighted, and undirected network with 200 nodes, with the weight of each edge determined by the Pearson correlation coefficient between the time series of its corresponding pair of nodes. We used the FC matrix of each of the 225 subjects to construct the FCNs, using a two-step procedure.

First, we determined the maximum spanning tree (MST) of the FC matrix using Kruskal's algorithm (Kruskal, 1956). The MST for a weighted graph with *n* nodes is an acyclic graph with (*n*−1) edges which includes all the nodes of the graph. Using an MST-based filtering approach ensures that the resulting FCN is a connected graph and it captures the highly correlated nodes from the FC matrix.

Second, we used a sparsity-based thresholding procedure, wherein edges from the FC matrix were iteratively added to the MST in decreasing order of their correlation values until we generated a network with the desired edge density. We binarized the thresholded network by removing the edge weights in order to obtain an unweighted, undirected and connected FCN with desired edge density (Achard and Bullmore, 2007; Rudie et al., 2013). Using a sparsity-based filtering approach ensures that the resulting FCNs for different subjects have the same number of edges, which enables a direct mathematical comparison of discrete Ricci curvatures and other network properties across subjects (Bassett et al., 2012; Rudie et al., 2013; Xu et al., 2016). We remark that such a two-step procedure with MST followed by sparsity-based thresholding was used previously by Achard et al. (2012) and Elumalai et al. (2022) to construct resting-state FCNs from FC matrices.

In this work, we computed discrete Ricci curvatures and other network properties over the range of edge densities 0.02–0.5 or 2–50% edges, with an increment of 0.01 or 1% edges (Rudie et al., 2013; Itahashi et al., 2014; Harlalka et al., 2018). Therefore, we constructed 49 FCNs for each of the 225 subjects from the MPI-LEMON dataset. We have made all the 200 × 200 FCNs for 225 subjects across 49 edge densities or thresholds publically available via a GitHub repository: https://github.com/asamallab/Curvature-FCN-Aging.

### 2.5. Discrete Ricci curvatures

Each FCN generated in our work can be represented as an unweighted and undirected graph or network *G* = (*V, E*), where *V* is the set of vertices or nodes in *G* and *E* is the set of edges or links in *G*. Classically, Ricci curvature is defined on tangent vectors on smooth manifolds (Jost, 2017), whereas for discrete objects such as networks, Ricci curvature is naturally defined on the edges. While both FRC (Forman, 2003; Sreejith et al., 2016) and ORC (Ollivier, 2007) are discrete analogs of the classical Ricci curvature, the definitions for FRC and ORC are non-equivalent, and therefore, the two discrete versions capture distinct properties of the classical Ricci curvature. Notably, FRC captures the geodesic dispersal property of the classical Ricci curvature whereas ORC captures the volume growth property of the classical Ricci curvature (Samal et al., 2018). Below, we present definitions of the two notions of discrete Ricci curvature, specific to an unweighted and undirected network, employed in our work.

#### 2.5.1. Forman-Ricci curvature

Forman (2003) introduced a discretization of Ricci curvature for a particular class of geometric objects known as *CW complexes*, which are widely studied within the field of algebraic topology. Some of us adapted the notion of Forman-Ricci curvature (FRC) to undirected graphs (Sreejith et al., 2016), which are equivalent to 1-dimensional CW complexes. However, a remarkable property of Forman's discretization is that it can be defined for CW complexes in any arbitrary number of dimensions, which allows for a rigorous means to account for higher-order interactions in complex systems. Notably, we utilized this property in a subsequent work (Samal et al., 2018) and defined a modified version of FRC, also known as *augmented* FRC. Formally, consider a graph *G* where weights are assigned to vertices, edges and triangular faces (that is, cycles of length 3). We refer to a vertex, an edge or a triangular face in *G* as a *cell*. Every cell in *G* can be assigned with a *dimension*. Specifically, vertices have dimension 0, edges have dimension 1 and triangles have dimension 2. The augmented FRC of an edge *e* in *G* can then be defined as


$$\begin{array}{l} F(e)=w_{e}[\left(\sum_{e<f}\frac{w_{e}}{w_{f}}+\sum_{v<e}\frac{w_{v}}{w_{e}}\right)\\ \;-\sum_{\hat{e}∥e}\left|\sum_{\hat{e},e<f}\frac{\sqrt{w_{e}\cdot w_{\hat{e}}}}{w_{f}}-\sum_{v<\hat{e},e}\frac{w_{v}}{\sqrt{w_{e}\cdot w_{\hat{e}}}}\right|] \end{array}$$

where *w*_*e*_ is the weight of edge *e*, *w*_*v*_ is the weight of vertex *v*, *w*_*f*_ is the weight of triangular face *f*.

For any two cells σ and τ in *G*, σ <τ denotes that σ is contained in τ. In other words, σ is a *lower dimensional* cell of τ. Specifically, for an edge *e* and a triangular face *f*, *e*<*f* means that the triangular face *f* contains the edge *e* as one of its sides. Moreover, for a vertex *v* and an edge *e*, *v*<*e* means that the edge *e* is anchored on the vertex *v*.

Any two cells with the same dimension can either share a higher dimensional cell or a lower dimensional cell. For example, two vertices *v*_1_ and *v*_2_ share an edge *e* if the edge *e* connects the vertices *v*_1_ and *v*_2_. On the other hand, two edges *e*_1_ and *e*_2_ share a vertex *v* if both *e*_1_ and *e*_2_ are anchored at *v*. Finally, two edges *e*_1_ and *e*_2_ share a triangular face *f*, if *e*_1_ and *e*_2_ are the sides of the triangle *f*.

Two cells of the same dimension are said to be *parallel* if they either share a higher dimensional cell or a lower dimensional cell, but not both. For example, two edges *e*_1_ and *e*_2_ are parallel by definition if they belong to the same triangular face or if they are anchored on the same vertex, but not both. ê∥*e* denotes the set of edges that are parallel to the edge *e*.

Augmented FRC accounts for cycles of length 3 in a graph while neglecting cycles of length 4 or higher. We refer the reader to Samal et al. (2018) for a systematic investigation of augmented FRC. If the graph *G* is unweighted, then all the vertices, edges, and triangular faces are assigned weight equal to 1, and the augmented FRC reduces to the following simple combinatorial expression


$$\begin{array}{l} \text{F}\left(e\right)=4-deg\left(i\right)-deg\left(j\right)+3\times tri\left(e\right) \end{array}$$

where *deg*(*i*) and *deg*(*j*) are the degrees of nodes *i* and *j*, respectively, that are anchored to the edge *e* and *tri*(*e*) is the number of triangular faces that contain the edge *e*. We refer to augmented FRC simply as FRC throughout the main text. Intuitively, FRC quantifies the extent of the spread of information around the ends of an edge in a network. A more negative value of FRC of an edge indicates a higher amount of information spread around its ends.

#### 2.5.2. Ollivier-Ricci curvature

For an edge *e* between nodes *i* and *j* in an unweighted and undirected graph *G*, ORC is measured by comparing the minimal cost of transporting a mass distribution over the neighbors of *i* and *j* with the distance between *i* and *j* itself (Ollivier, 2007; Ni et al., 2015). Formally,


$$\begin{array}{l} \text{O}\left(e\right)=1-\frac{W_{1}\left(m_{i},m_{j}\right)}{d\left(i,j\right)}, \end{array}$$

where *m*_*i*_ and *m*_*j*_ are the discrete probability measures defined on nodes *i* and *j*, respectively, and *d*(*i, j*) is the distance between *i* and *j*. Since *G* is an unweighted graph, *d*(*i, j*) is equal to the number of edges contained in the shortest path connecting *i* and *j*. *W*_1_ is the transportation distance between *m*_*i*_ and *m*_*j*_, also known as the Wasserstein distance (Vaserstein, 1969), which is defined as


$$\begin{array}{l} W_{1}\left(m_{i},m_{j}\right)=\underset{\mu_{i,j}\in \prod \left(m_{i},m_{j}\right)}{\inf}\underset{\left(i^{\prime },j^{\prime }\right)\in V\times V}{\sum }d\left(i^{\prime },j^{\prime }\right)\mu_{i,j}\left(i^{\prime },j^{\prime }\right), \end{array}$$

where ∏(*m*_*i*_, *m*_*j*_) is the set of probability measures μ_*i, j*_ that satisfy


$$\begin{array}{l} \underset{j^{\prime }\in V}{\sum }\mu_{i,j}\left(i^{\prime },j^{\prime }\right)=m_{i}\left(i^{\prime }\right),\underset{i^{\prime }\in V}{\sum }\mu_{i,j}\left(i^{\prime },j^{\prime }\right)=m_{j}\left(j^{\prime }\right). \end{array}$$

The above equation gives all the possible transportations of measure *m*_*i*_ to *m*_*j*_. The Wasserstein distance *W*_1_(*m*_*i*_, *m*_*j*_) is the minimal cost of transporting *m*_*i*_ to *m*_*j*_. The probability distribution *m*_*i*_ is considered to be uniform over the neighboring nodes of *i* (Lin et al., 2011). Note that the computation of ORC can be formulated as a linear programming problem (Ni et al., 2015).

### 2.6. Global and node-level network analyses

For each of the 225 subjects in the MPI-LEMON dataset included in this study, we generated 49 unweighted and undirected FCNs with varying edge densities from 2 to 50% with an increment of 1%. Subsequently, we compared the global or whole-brain characteristics of the FCNs, at each edge density, across the two groups (young and elderly) using FRC and ORC. Specifically, we computed the average FRC of edges and average ORC of edges, for each of the 49 networks, for each of the 225 subjects included in this study. Additionally, we computed eight other global network measures namely, clique number, average clustering coefficient, global efficiency (Latora and Marchiori, 2001), average node betweenness centrality, average local efficiency (Latora and Marchiori, 2001), average shortest path length, modularity (Blondel et al., 2008), and assortativity. We provide the definitions of theses global network measures used to characterize the FCNs in the Supplementary material.

Next, we compared the node or region-level characteristics of the FCNs, at each edge density, across the young and elderly groups. Specifically, we computed the node FRC and node ORC for the 200 nodes, for each of the 49 networks, for each of the 225 subjects included in this study. The notion of node Ricci curvature is analogous to the notion of scalar curvature in Riemannian geometry (Jost, 2017) and is computed as the sum of the Ricci curvatures for the edges incident on a given node (Samal et al., 2018).

The computer codes used to compute the global and node-level network characteristics including FRC and ORC are publicly available via a GitHub repository: https://github.com/asamallab/Curvature-FCN-Aging. The standard network measures were computed using the Python package NetworkX (Hagberg et al., 2008).

### 2.7. Neurosynth meta-analysis decoding

In order to determine the cognitive and behavioral relevance of the results from node-level comparisons across the young and elderly groups, we used Neurosynth meta-analysis decoding (Yarkoni et al., 2011; Williams et al., 2021). First, we used the Neurosynth meta-analysis tool to find terms related to cognition, perception and behavior corresponding to the centroid coordinates of each of the 200 Schaefer ROIs. Second, we assigned each of the nodes or ROIs with significant between-group differences in FRC or ORC to their respective RSN, as defined by the Schaefer atlas. The ROIs were assigned to one of seven such RSNs, since the Schaefer atlas is divided into seven RSNs. Third, for a given RSN, we computed the number of occurrences of the terms associated with the significant ROIs within that RSN. Fourth, we determined the statistical significance of the number of occurrences of the different terms associated with each RSN. Note that the above steps were performed separately for each RSN.

### 2.8. Statistical analyses

We employed a two-tailed two-sample *t*-test to estimate the differences in average edge curvatures and other global network measures between the young and elderly groups throughout the 49 FCN densities in the range 2–50% considered in this work. In order to estimate the between-group differences in node curvatures, we first computed the area under the curve (AUC) for the FRC or ORC of a given node across the 49 edge densities (Achard and Bullmore, 2007; Itahashi et al., 2014). Subsequently, we employed a two-tailed two-sample *t*-test on the AUCs of the discrete curvatures for each of the 200 nodes of the FCNs.

In order to determine the statistical significance of the number of occurrences of each term in the Neurosynth meta-analysis decoding, we first computed the number of occurrences of the same terms corresponding to equal number of randomly selected nodes (surrogate ROIs). For example, if we identified 66 nodes with significant between-group differences in FRC, then we randomly selected a set of 66 nodes out of the 200 nodes in the Schaefer atlas. We obtained a null distribution for the number of occurrences of each term by generating 1,000 sets of randomly selected nodes. Subsequently, we calculated the *z*-score corresponding to the number of occurrences of each term associated with the subset of original ROIs. Finally, we converted the *z*-scores to *p*-values, assuming a normal distribution (Williams et al., 2021; Elumalai et al., 2022).

In order to correct for multiple comparisons and control the occurrence of false positives, we employed a false discovery rate (FDR) correction (Benjamini and Hochberg, 1995) to adjust the *p*-values for each of the statistical tests described above. We set the threshold α for these FDR corrections to 0.05. These statistical tests were performed using Python packages SciPy (Virtanen et al., 2020) and statsmodels (Seabold and Perktold, 2010). No covariates were used in the statistical analyses.

### 2.9. Correlation of node curvatures with cognitive and behavioral scores

Neurosynth meta-analysis decoding revealed cognitive and behavioral domains associated with those brain regions whose node curvatures were different between young and elderly participants. We performed a correlation analysis to determine the extent to which curvatures of individual brain regions could account for variation in performance on tests measuring ability in cognitive or behavioral domains linked to those regions. Specifically, if the Neurosynth meta-analysis for a given RSN revealed a particular cognitive or behavioral domain, then we acquired all the phenotypic test scores associated with that domain for each of the 225 subjects in the MPI-LEMON dataset. Note that the MPI-LEMON dataset contains detailed information on 6 cognitive tests and 21 questionnaires related to emotional behavior, personality traits and tendencies, eating behavior, and addictive behavior. This data can be accessed via the links https://ftp.gwdg.de/pub/misc/MPI-Leipzig_Mind-Brain-Body-LEMON/ or http://fcon_1000.projects.nitrc.org/indi/retro/MPI_LEMON.html.

Next, we computed Spearman's correlation between the node FRC (or node ORC) of all the significant regions in that RSN and the phenotypic scores provided in each of the cognitive or behavioral tests. We used Spearman rather than Pearson correlation since some of the test scores are expressed on an ordinal rather than continuous scale. Finally, we performed FDR correction to adjust the *p*-values of the estimated correlations. FDR correction was applied to *p*-values of each cognitive or behavioral test, separately. Note that if a particular cognitive or behavioral test had multiple phenotypic scores, then the FDR correction was applied across all the phenotypic scores within that test.

### 2.10. Literature search of non-invasive brain stimulation studies in healthy elderly individuals

We performed a literature search on PubMed to identify scientific papers reporting the effect of three non-invasive brain stimulation techniques namely, tDCS, tACS, and TMS on motor performance in healthy elderly individuals. Then, we assessed the results reported in these papers to identify brain regions that show evidence for improvements in motor performance of elderly individuals after non-invasive stimulation. The following search query was used in PubMed: [(transcranial magnetic stimulation) OR (TMS) OR (transcranial direct current stimulation) OR (tDCS) OR (transcranial alternating current stimulation) OR (tACS)] AND [(healthy old) OR (healthy elderly) OR (healthy aging) OR (healthy aging)] AND [(motor function) OR (motor performance) OR (motor skill) OR (movement)]. This PubMed search was performed in October 2022, and returned a list of 1,659 articles.

We followed a three-stage procedure to identify relevant articles from the list of 1,659 articles returned by the PubMed search (see Supplementary Figure 1). First, we checked all the meta-analysis papers studying the effects of tDCS, tACS, or TMS on motor performance in elderly individuals (Summers et al., 2016; Patel et al., 2019; Lee et al., 2021; Pino-Esteban et al., 2021) to identify articles that were missing from the list of 1,659 articles. Second, we filtered the relevant articles from the list of 1,659 articles based on title and abstract. Third, we classified the articles according to the non-invasive stimulation technique employed (tDCS, tACS, or TMS) and checked the full text of the articles for relevance. We used the following inclusion criteria to judge article relevance: (1) studies on healthy elderly individuals, (2) studies that employ non-invasive brain stimulation, namely tDCS, tACS, and TMS, (3) studies that analyze the effect of non-invasive stimulation techniques on motor performance, as measured during movement-related tasks such as finger tapping tasks (FTT), sequence learning tasks and serial reaction time tasks (SRTT), and their variants, and (4) studies that are peer-reviewed. We used the following exclusion criteria to judge article relevance: (1) review articles, (2) articles presented in languages other than English, (3) studies that did not employ non-invasive stimulation, (4) studies that investigate new protocols for brain stimulation, (5) studies on diseased populations, including patients with Alzheimer's Disease, Parkinson's Disease or Stroke, (6) studies on non-human species, e.g., rats, (7) studies that employed non-invasive stimulation on children or young adults, (8) studies that analyze the effect of non-invasive stimulation on domains other than motor performance, and (9) studies that analyze the effect of non-invasive stimulation on neuronal activity, neuroplasticity or neurophysiological processes. A given article was considered as relevant only if all the inclusion criteria were satisfied, whereas it was considered as not relevant if at least one exclusion criterion was satisfied.

The above procedure resulted in 36 eligible articles that employed tDCS, three eligible articles that employed tACS, and two eligible articles that employed TMS in their experiments. We extracted the following data from the eligible articles: PMID, author, publication year, participant information (which includes number of participants and mean age of the participants), type of stimulation (for example, anodal tDCS, cathodal tDCS, bilateral tDCS, rTMS), target brain region, stimulation parameters (such as stimulation intensity for tDCS and tACS, pulses per session and inter-train interval for TMS, number of sessions, and treatment duration), outcome measures used to assess motor performance after stimulation, results of stimulation, and adverse effects of stimulation on experimental group or control group (if applicable). Supplementary Table 2 provides the data extracted from the tDCS, tACS, and TMS studies, respectively. From the above data, we combined information about the target region, outcome measures and results of tDCS/tACS/TMS stimulation as measured using the outcome measures in each study to identify the set of target regions whose non-invasive stimulation resulted in improved motor performance of healthy older individuals. These target regions were used in a subsequent analysis to determine whether FRC or ORC can identify brain regions whose non-invasive stimulation shows evidence for improvements in motor performance of healthy older individuals.

### 2.11. Determining overlap between brain regions with age-related differences in curvature and target regions in non-invasive stimulation studies

Next, we performed a systematic comparison of the age-related differences in discrete Ricci curvatures with results from previous non-invasive stimulation studies. However, note that the target regions in our collected list of non-invasive brain stimulation experiments correspond to the ROIs in the Brodmann atlas (Strotzer, 2009), whereas the brain regions with age-related differences in FRC or ORC correspond to the ROIs in the Schaefer 200 atlas. Therefore, in order to enable such a comparison, we merged the two atlases together and subsequently determined the overlap between brain regions with age-related differences in curvature and target regions of non-invasive brain stimulation experiments. More precisely, we employed the following two-step procedure. First, we mapped each Schaefer ROI to a corresponding Brodmann ROI. The mapping was performed using the MRIcron tool (Rorden et al., 2007) by identifying the Brodmann area that contains the MNI centroid coordinates of a given Schaefer ROI. We remark that a similar mapping approach was employed in our previous study (Elumalai et al., 2022) where some of us applied discrete Ricci curvatures to identify brain regions related to atypical functional connectivity in ASD. Second, we determined the set of Brodmann ROIs corresponding to the target regions in non-invasive brain stimulation experiments that show evidence for improvements in motor performance in healthy elderly. Third, we identified the set of Schaefer ROIs that were mapped to these Brodmann ROIs. Fourth, we identified the Schaefer ROIs within this set that also showed significant age-related differences in FRC or ORC.

## 3. Results

In this work, we employed two geometry-inspired graph measures namely, FRC and ORC, to investigate the whole-brain and region-level differences in resting state FCNs during healthy aging. We preprocessed raw rs-fMRI images of 153 healthy, young individuals in the age range 20–35 years and 72 healthy, elderly individuals in the age range 59–77 years from the MPI-LEMON dataset. Next, we used the Schaefer atlas (Schaefer et al., 2018) to parcellate the preprocessed rs-fMRI scan of each subject into 200 ROIs, and calculated the Pearson correlation coefficient between all pairs of ROI time series, yielding a 200 × 200 FC matrix. Finally, we generated 49 FCNs for each subject across the range of edge densities 0.02–0.5 or 2–50% edges, with an increment of 0.01 or 1% edges using a two-step edge filtering approach comprising MST and sparsity-based thresholding. The detailed methodology to construct and analyze the FCNs is described in Section 2.

### 3.1. Whole-brain differences in functional connectivity networks

**Figure 2:**
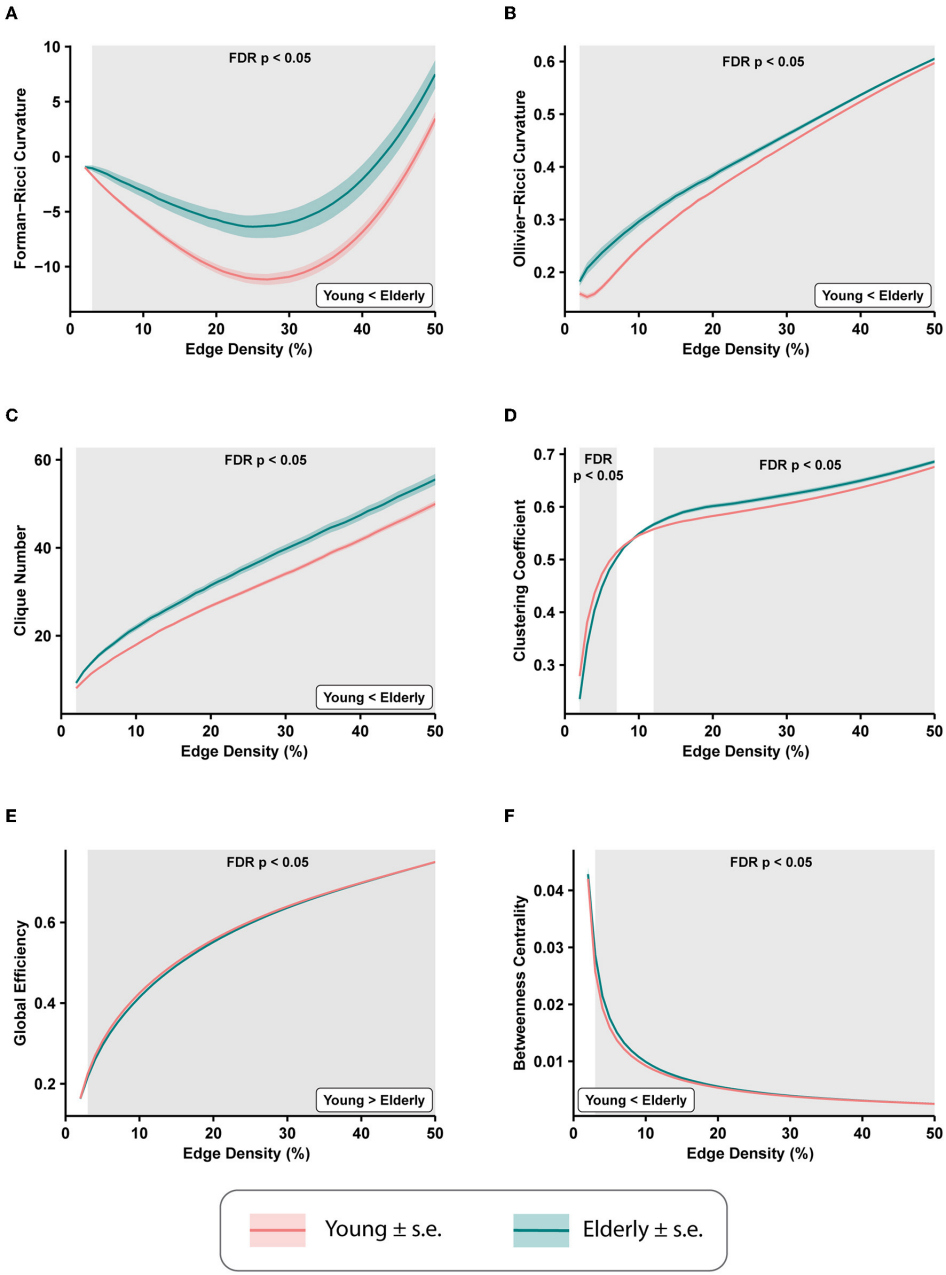
Differences in average edge curvatures and standard global network measures across the functional connectivity networks (FCNs) of 153 young individuals and 72 elderly individuals from the MPI-LEMON dataset. The differences are reported for FCNs over the range of edge densities 0.02 (i.e., 2% edges) and 0.5 (i.e., 50% edges), with an increment of 0.01 (i.e., 1% edges). The shaded regions in each plot correspond to the edge densities where the between-group differences are statistically significant (*p* < 0.05, FDR-corrected). **(A)** Average Forman-Ricci curvature (FRC) of edges is higher in elderly individuals over edge densities 3–50%. **(B)** Average Ollivier-Ricci curvature (ORC) of edges is higher in elderly individuals over the entire range of edge densities considered (2–50%). **(C)** Clique number is higher in elderly individuals over all edge densities 2–50%. **(D)** Average clustering coefficient is higher in young individuals over edge densities 2–7%, and higher in elderly individuals over edge densities 12–50%. **(E)** Global efficiency is reduced in elderly individuals over edge densities 3–50%. **(F)** Average node betweenness centrality is higher in elderly individuals over edge densities 3–49%.

We computed the average edge FRC and average edge ORC for the 49 FCNs across edge densities 2–50% for each of the 225 subjects to evaluate the age-related brain-wide changes in FCNs between the healthy young and healthy elderly groups. Subsequently, we applied a two-tailed two-sample *t*-test with FDR correction (see Section 2) to estimate the differences in average edge curvatures between the young and elderly groups at each value of edge density. Figures 2A, B show the differences in average edge FRC and average edge ORC, respectively, between the young and elderly individuals over the range of edge densities 2–50%. We found that the elderly group had significantly higher values of average edge FRC (*p* < 0.05, FDR-corrected) over the range of edge densities 3–50% (Figure 2A). Similarly, we found that the elderly group had significantly higher values of average edge ORC over the entire range of edge densities 2–50% considered in this study (Figure 2B).

We further compared eight other global network measures to evaluate brain-wide changes in FCNs of young and elderly individuals. The eight global network measures include clique number, average clustering coefficient, global efficiency, average node betweenness centrality, average local efficiency, shortest path length, modularity, and assortativity. Out of the eight measures, clique number, node betweenness centrality, and assortativity have not been applied in previous network-based analyses of rs-fMRI scans in healthy aging. First, we found that the clique number is significantly higher in elderly group (*p* < 0.05, FDR-corrected) over all edge densities considered in this study (Figure 2C). Second, we found average clustering coefficient is significantly higher in the young group at edge densities 2–7%, whereas it is significantly higher in the elderly group at edge densities 12–50% (Figure 2D). This change in the directionality of the differences in clustering coefficient with increasing edge densities suggests that highly correlated nodes or ROIs tend to cluster in young individuals whereas weakly correlated nodes tend to cluster in elderly individuals. Notably, the group differences in clustering coefficient observed in the present work are consistent with previous studies that have used clustering coefficient to compare the global changes in resting state FCNs in healthy aging cohorts (Sala-Llonch et al., 2014; Li et al., 2016). Third, we found that global efficiency of the FCNs is significantly reduced in elderly individuals over the edge density range 3–50% (Figure 2E), and our results are consistent with previous graph-theoretic rs-fMRI studies (Achard and Bullmore, 2007; Sala-Llonch et al., 2014; Song et al., 2014; Geerligs et al., 2015; Li et al., 2016) in healthy aging populations. Fourth, we found that average node betweenness centrality of the FCNs is significantly higher in elderly individuals over the edge density range 3–49% (Figure 2F).

Fifth, we found that average local efficiency is significantly higher in the FCNs of the young group compared to the elderly group over the edge density range 1–12%, and significantly higher in the elderly group over the edge density range 19–49% (Supplementary Figure 2). Notably, the differences in average local efficiency observed in the present work are consistent with previous graph-theoretic rs-fMRI studies (Achard and Bullmore, 2007; Song et al., 2014; Geerligs et al., 2015) in healthy aging populations. Sixth, we found that average shortest path length is significantly higher in the elderly group over the edge density range 3–50% (Supplementary Figure 2), and these differences are consistent with previous graph-theoretic rs-fMRI studies (Sala-Llonch et al., 2014) in healthy aging populations. Seventh, we found that modularity is significantly reduced in elderly individuals over the range of edge densities 2–9% (Supplementary Figure 2), and these differences are consistent with results from previous graph-theoretic rs-fMRI studies (Song et al., 2014; Geerligs et al., 2015) in healthy aging populations. Eighth, we found that assortativity is reduced in the elderly group, but the differences are not significant (as suggested by *p*>0.05, FDR-corrected).

Supplementary Table 3 lists the FDR-corrected *p*-values corresponding to the group-wise comparisons of discrete Ricci curvatures and standard network measures across all edge densities 2–50% considered in this study.

### 3.2. Region-level differences in functional connectivity networks

As reported in the previous subsection, we found that both FRC and ORC show significant differences between the resting state FCNs of young and elderly individuals at the whole-brain level. Subsequently, we determined how the global differences in discrete Ricci curvatures are distributed across the 200 nodes or ROIs in the brain as defined by the Schaefer atlas (see Section 2). First, for each of the 225 subjects, we computed node FRC and node ORC for the 200 nodes in the 49 FCNs across the range of edge densities 2–50%. Second, in order to determine the nodes with significant between-group differences in node Ricci curvatures in the FCNs of young and elderly individuals, we computed the Area Under Curve (AUC) of the node curvature values across the 49 edge densities for each of the 200 nodes, and compared these AUCs for each node using a two-tailed two-sample *t*-test with FDR correction.

**Figure 3:**
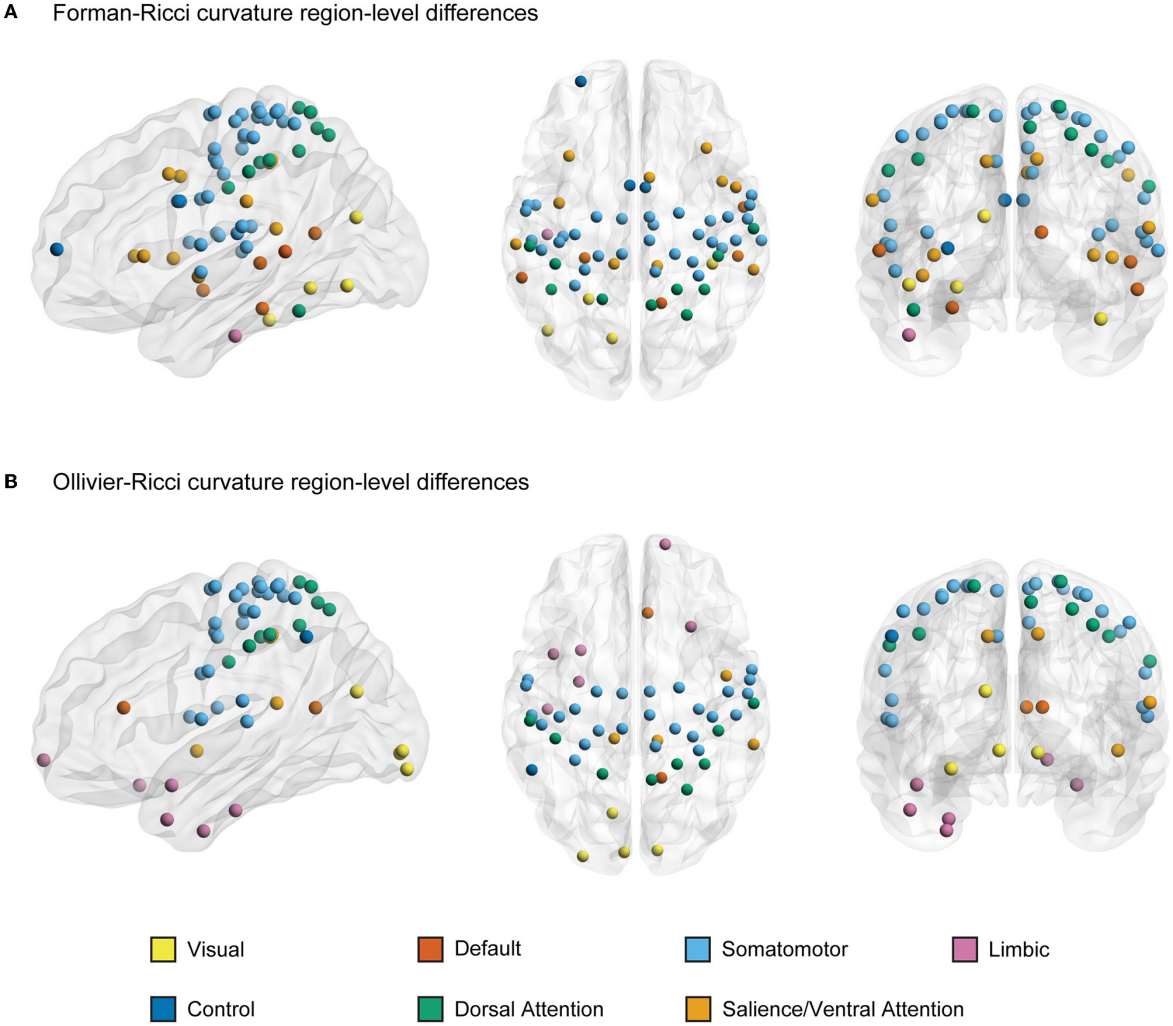
A visual representation of nodes or regions in the brain that show significant differences in discrete Ricci curvatures between the functional connectivity networks (FCNs) of young and elderly individuals (*p* < 0.05, FDR corrected). **(A)** 66 regions that show significant differences in Forman-Ricci curvature (FRC) of the nodes in the FCNs of the young and elderly groups. All the 66 regions display higher values of node FRC for elderly individuals compared to young individuals. **(B)** 53 regions that show significant differences in Ollivier-Ricci curvature (ORC) of the nodes in the FCNs of the young and elderly groups. 41 out of these 53 regions display higher values of node ORC in elderly individuals and the remaining 12 ROIs display higher values of node ORC in young individuals. The nodes are specified by the Schaefer atlas, and color-coded as per the 7 resting state networks (RSNs) listed in the figure legend. This figure was created using BrainNet Viewer (Xia et al., 2013). Supplementary Table 5 lists the FDR corrected *p*-values corresponding to curvature-related differences in each region.

Figures 3A, B illustrate the nodes or ROIs that display significant between-group differences (*p* < 0.05, FDR-corrected) in FRC and ORC, respectively, of the FCNs of young and elderly individuals. We identified 66 ROIs with significant differences in FRC and 53 ROIs with significant differences in ORC across the young and elderly groups. We found 42 ROIs to be identified by both FRC and ORC. We found that the significant ROIs identified by both FRC and ORC are spread across the 7 RSNs. However, the ROIs with significant differences in node FRC are concentrated in 3 RSNs namely, somatomotor network (33 ROIs), salience/ventral attention network (10 ROIs) and dorsal attention network (10 ROIs). The ROIs with significant differences in node ORC are concentrated in 2 RSNs namely, somatomotor network (27 ROIs) and dorsal attention network (9 ROIs). Supplementary Table 4 lists the ROIs with significant between-group differences as identified by FRC and ORC, partitioned across the 7 RSNs.

Further, we determined the directionality of the significant differences in node FRC and node ORC between the young and elderly individuals across each of the 200 nodes or ROIs. Recall that at the whole-brain level, both FRC and ORC displayed higher values in elderly individuals compared to young individuals. We found that all the 66 ROIs with significant between-group differences in node FRC display higher values of node FRC for elderly individuals compared to young individuals. Moreover, we found that 41 out of the 53 ROIs with significant between-group differences in node ORC display higher values of node ORC in elderly individuals and the remaining 12 ROIs display higher values of node ORC in young individuals. Supplementary Table 5 lists the AUCs of the node FRC and node ORC for each of the 200 nodes averaged across the young and elderly groups, along with the FDR-corrected *p*-values.

### 3.3. Behavioral and cognitive relevance of region-level differences

As reported in the previous subsection, we identified 66 nodes or ROIs that show significant between-group differences in FRC (Figure 3A) and 53 ROIs that show significant between-group differences in ORC (Figure 3B) across the young and elderly groups. We partitioned the set of significant ROIs into 7 subsets based on their respective RSNs as defined by the Schaefer atlas. Subsequently, we determined the cognitive domains associated with the significant ROIs in each RSN using Neurosynth meta-analysis (see Section 2). For nodes identified by FRC, we limited the Neurosynth analysis to somatomotor network, salience/ventral attention network, and dorsal attention network. For nodes identified by ORC, we limited the Neurosynth analysis to somatomotor network and dorsal attention network. We chose the above-mentioned RSNs since the significant ROIs identified by FRC or ORC are mainly concentrated within these RSNs. Moreover, the sets of significant ROIs in these RSNs are nearly bilaterally symmetrical.

**Figure 4:**
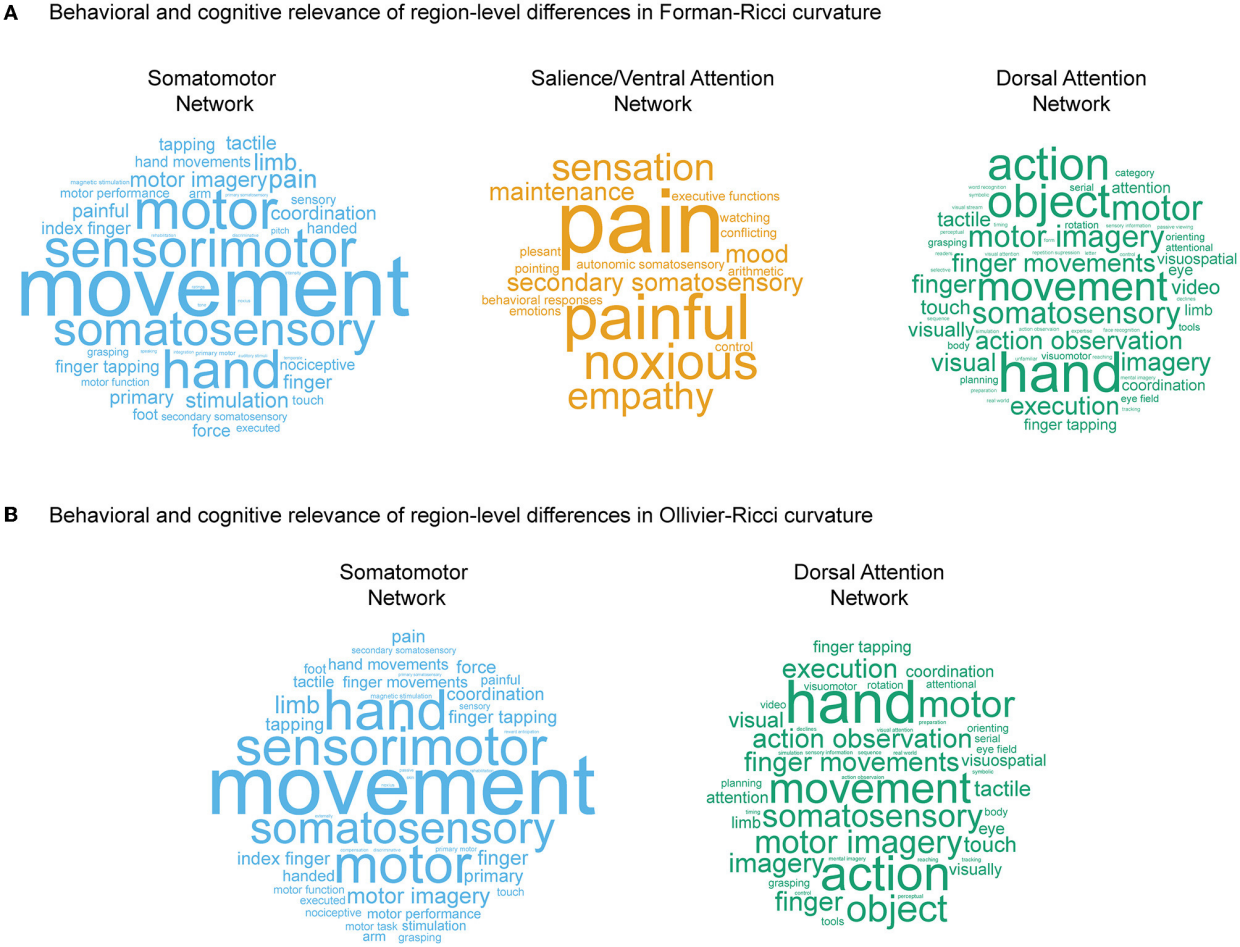
Behavioral and cognitive relevance of age-related region-level differences in curvatures. **(A)** Word clouds displaying cognitive and behavioral terms associated with brain regions having age-related differences in values of Forman-Ricci curvature (FRC), in three RSNs, namely somatomotor network, salience/ventral attention network, and dorsal attention network. **(B)** Word clouds displaying cognitive and behavioral terms associated with brain regions having age-related differences in values of Ollivier-Ricci curvature (ORC), in two RSNs, namely somatomotor network and dorsal attention network. The size of the terms in each word cloud is proportional to their frequency of occurrence. Note that size of the terms in each word cloud are scaled separately, and thus, frequencies of occurrence cannot be compared across word clouds. The word clouds in this figure are generated using https://www.wordclouds.com.

Figure 4A shows the word clouds highlighting the behavioral relevance of the significant brain regions associated with three main RSNs identified by FRC, and Figure 4B shows the word clouds for the significant brain regions associated with two main RSNs identified by ORC. Supplementary Table 6 lists the terms associated with the significant regions identified by FRC and ORC across all 7 RSNs. For the nodes identified by FRC, the word cloud for somatomotor network shows terms associated with movement. For salience/ventral attention network, we find terms associated with affective and somatosensory processing. For dorsal attention network, we find terms associated with movement. For the nodes identified by ORC, the word clouds for both somatomotor network and dorsal attention network show terms associated with movement.

### 3.4. Correlation between curvatures and affective processing-related phenotypic test scores

The Neurosynth meta-analysis revealed that brain regions showing age-related FRC and ORC differences in somatomotor and dorsal attention networks are associated with movement, and brain regions showing age-related FRC differences in salience/ventral attention network are associated with affective and somatosensory processing (Figure 4). To do this, we performed a *post-hoc* correlation analysis to determine the extent to which node FRC and node ORC of the brain regions with age-related differences in a given RSN, could account for variation in phenotypic test scores corresponding to the cognitive or behavioral domains identified by the Neurosynth meta-analysis for that RSN.

We identified 8 tests related to affective processing, for each of the 225 subjects in the MPI-LEMON dataset, but did not find any tests related to movement or somatosensory processing. In the following, we list these tests related to affective processing: (i) Cognitive Emotion Regulation Questionnaire (CERQ), (ii) Coping Orientations to Problems Experienced (COPE), (iii) Emotion Regulation Questionnaire (ERQ), (iv) Measure of Affect Regulation Style (MARS), (v) Perceived Stress Questionnaire (PSQ), (vi) State-Trait-Angstinventar (STAI-G-X2), (vii) Trait Emotional Intelligence Questionnaire—Short Form (TEIQue-SF), and (vii) Trierer Inventar zum Chronischen Stress (TICS).

We found that node FRC showed significant correlation (*p* < 0.05, FDR-corrected) only with the TICS phenotypic scores. TICS contains 9 scores that are designed to measure the level of chronic stress that an individual experiences across 9 subdomains including work overload, social overload, pressure to perform, work discontent, excessive demands from work, lack of social recognition, social tension, social isolation, and chronic worrying (Schulz et al., 2004; Petrowski et al., 2020). Further, TICS contains a separate score called Short Screening Scale for Chronic Stress (SSCS) which was designed as brief chronic stress instrument for applied research and practitioners. Hence, 10 scores are recorded in the TICS test.

**Figure 5:**
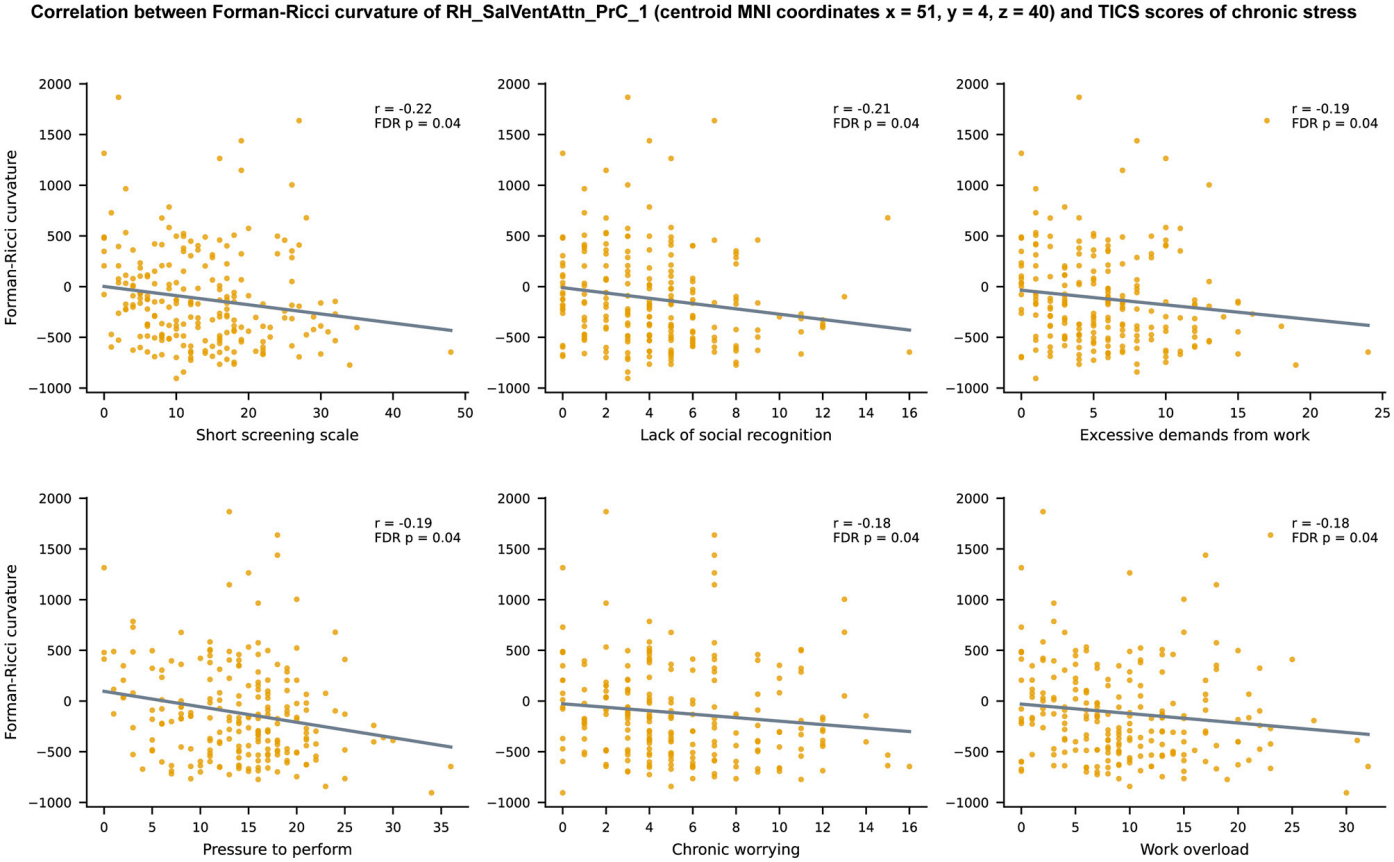
Scatter plots depicting the relationship between Forman-Ricci curvature (FRC) of the brain region RH_SalVentAttn_PrC_1 and 6 TICS scores of chronic stress namely, Short Screening Scale for Chronic Stress (SSCS), lack of social recognition, excessive demands from work, pressure to perform, chronic worrying, and work overload. Note that only the scatter plots corresponding to significant correlations *r* between FRC of RH_SalVentAttn_PrC_1 and TICS scores (*p* < 0.05, FDR corrected) are shown in this figure. Each plot displays a line describing the linear relationship between FRC of RH_SalVentAttn_PrC_1 and the corresponding TICS score, estimated using the least squares method. The brain region is named according to the labeling scheme provided in the Schaefer atlas (Schaefer et al., 2018).

Since 10 brain regions in ventral attention network revealed node FRC age-related differences, we performed 10 × 10 = 100 FRC—TICS correlations, out of which 17 correlations were statistically significant (*p* < 0.05, FDR-corrected). The 17 statistically significant correlations were spread across seven brain regions, with node FRC of RH_SalVentAttn_PrC_1 being correlated to as many as 6 TICS scores (Figure 5), while the other statistically significant correlations were distributed between RH_SalVentAttn_Med_2, RH_SalVentAttn_Med_1, RH_SalVentAttn_FrOperIns_4, RH_SalVentAttn_TempOccPar_2, LH_SalVentAttn_Med_2, and LH_SalVentAttn_FrOperIns_3 (Supplementary Figure 3). All the 17 statistically significant FRC—TICS correlations were negative, ranging between −0.22 and −0.18. Hence, we find that higher node FRC values are associated with lower levels of chronic stress.

### 3.5. Comparison of region-level differences in curvatures with tDCS/tACS/TMS data

The Neurosynth meta-analysis performed in this work revealed that brain regions with age-related FRC and ORC differences in somatomotor and dorsal attention networks are associated with movement. Movement is known to be impaired with age (Seidler et al., 2010; Cirillo, 2021). Subsequently, we performed a *post-hoc* analysis to determine the overlap between the set of brain regions with age-related FRC and ORC differences in somatomotor and dorsal attention networks, and the set of brain regions whose stimulation with non-invasive techniques, e.g., tDCS, yielded improvement in motor function of elderly individuals. An overlap between these two sets of regions would provide additional evidence, apart from results from the Neurosynth meta-analysis, that FRC and ORC identify brain regions that are related to movement impairments in healthy elderly individuals. To perform this analysis, we first performed a literature search on PubMed to identify scientific papers reporting the effect of non-invasive brain stimulation on motor performance in healthy elderly individuals. The details of the PubMed search query are provided in Section 2. Supplementary Figure 1 summarizes the workflow we employed to collect and classify the eligible articles from the literature survey, as guided by preferred reporting items for systematic reviews and meta-analysis (PRISMA) (Moher et al., 2009). We used results reported in these eligible articles to identify those brain regions whose stimulation in elderly individuals using tDCS, tACS, or TMS yielded improvement in motor performance. Finally, we compared this set of brain regions to those with age-related FRC or ORC differences in somatomotor and dorsal attention networks.

We found that previous studies applying tDCS on healthy old adults reported significant improvements in motor performance upon stimulating 4 target regions namely, primary motor cortex (M1), dorsolateral prefrontal cortex (DLPFC), right supplementary motor area (SMA), and the cerebellum. Previous studies that applied tACS on healthy old adults reported significant improvements in motor performance upon stimulating 3 target regions namely, DLPFC, posterior parietal cortex (PPC), and left M1. Finally, previous studies that applied TMS on healthy old adults reported significant improvements in motor performance upon stimulating the left DLPFC. We refer readers to Supplementary Table 2 for details about the tDCS, tACS, and TMS studies.

**Figure 6:**
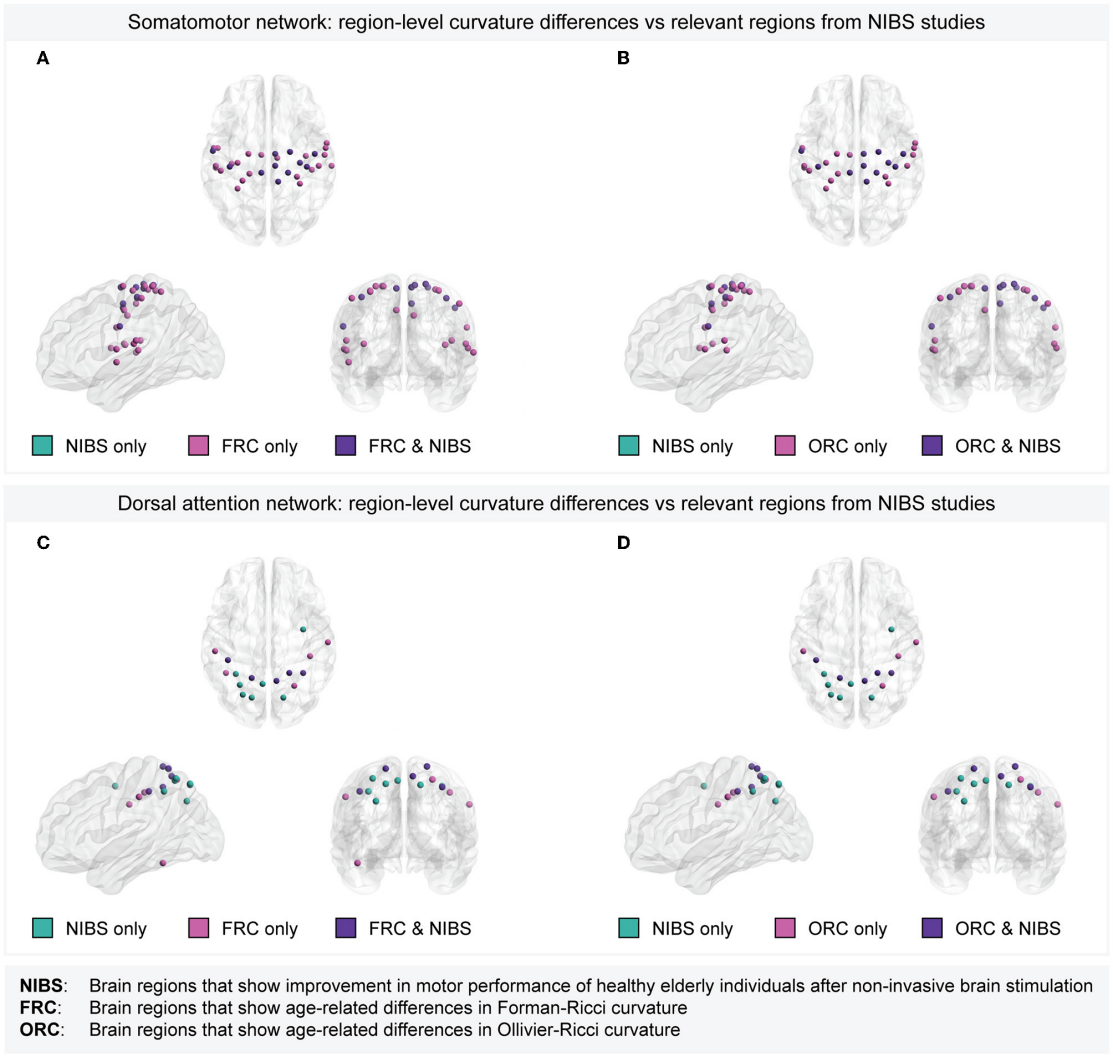
Overlap between brain regions with age-related curvature differences and target regions whose non-invasive stimulation resulted in improved motor performance of healthy elderly individuals. **(A)** Within the somatomotor network, overlap between the set of 33 Schaefer ROIs with age-related differences in Forman-Ricci curvature (FRC) and 11 Schaefer ROIs corresponding to the target regions used in tDCS/tACS/TMS experiments. All the 11 ROIs corresponding to the target regions show age-related differences in FRC. **(B)** Within the somatomotor network, overlap between the set of 27 Schaefer ROIs with age-related differences in Ollivier-Ricci curvature (ORC) and 11 Schaefer ROIs corresponding to the target regions used in tDCS/tACS/TMS experiments. All the 11 ROIs corresponding to the target regions show age-related differences in ORC. **(C)** Within the dorsal attention network, overlap between the set of 10 Schaefer ROIs with age-related differences in FRC and 12 Schaefer ROIs corresponding to the target regions used in tDCS/tACS/TMS experiments. Five out of the 12 ROIs corresponding to the target regions show age-related differences in FRC. **(D)** Within the dorsal attention network, overlap between the set of 9 Schaefer ROIs with age-related differences in ORC and 12 Schaefer ROIs corresponding to the target regions used in tDCS/tACS/TMS experiments. Five out of the 12 ROIs corresponding to the target regions show age-related differences in ORC. Note that the set of brain regions in each subfigure is partitioned into the following three subsets. Regions that are relevant according to non-invasive stimulation studies but do not show age-related curvature differences (labeled as “NIBS only”), regions that show age-related curvature differences but lack evidence from non-invasive stimulation studies (labeled as “FRC only” or “ORC only”), and regions that show both age-related curvature differences as well as relevance according to non-invasive stimulation studies (labeled as “FRC & NIBS” or “ORC & NIBS”). In **(A, B)**, there are no regions labeled “NIBS only” since all the regions in the somatomotor network that are relevant according to non-invasive stimulation studies also show age-related differences in FRC and ORC, respectively.

Note that the target regions stimulated in the previous tDCS, tACS, or TMS studies correspond to ROIs in the Brodmann atlas (Strotzer, 2009; Cieslik et al., 2013), whereas the ROIs in the present study are defined according to the Schaefer atlas. Thus, we mapped the ROIs in the Schaefer atlas to ROIs in the Brodmann atlas (Elumalai et al., 2022) (see Section 2) to enable systematic comparison of the age-related region-level differences in discrete Ricci curvatures with those target regions whose stimulating resulted in improved motor performance. Supplementary Table 7 lists the ROIs in the Brodmann atlas corresponding to each of the 200 ROIs in the Schaefer atlas. Across tDCS, tACS, and TMS, the four cortical target regions showing evidence for improvements in motor performance in healthy elderly individuals include M1 (Brodmann area 4), DLPFC (Brodmann areas 9 and 46; Cieslik et al., 2013), PPC (Brodmann areas 5, 7, 39, and 40; Whitlock, 2017), and right SMA (Brodmann area 6). These target regions map to 42 ROIs in the Schaefer atlas. Out of these 42 ROIs, 11 ROIs belong to the somatomotor network and 12 ROIs belong to the dorsal attention network. All the 11 ROIs in the somatomotor network whose stimulation resulted in improved motor performance in healthy older individuals also showed age-related differences in both FRC as well as ORC. Further, five out of the 12 ROIs in the dorsal attention network whose stimulation resulted in improved motor performance in healthy older individuals also showed age-related differences in both FRC as well as ORC. Figure 6 provides a visual illustration of the overlaps between the set of ROIs whose non-invasive stimulation resulted in improved motor performance in elderly, and the set of ROIs with age-related differences in FRC or ORC, within the somatomotor and dorsal attention networks. These results provide evidence that both FRC and ORC identify brain regions that are related to movement impairments in healthy elderly individuals.

## 4. Discussion

Prior to this work, the utility of discrete Ricci curvatures in characterizing alterations in FCNs related to healthy aging remained unexplored. In the present work, we apply two widely-used notions of discrete Ricci curvature, FRC and ORC, to study resting-state functional connectivity differences in 153 healthy young and 72 healthy old subjects from the MPI-LEMON dataset (Babayan et al., 2019). Raw rs-fMRI scans for each subject were processed using a uniform preprocessing pipeline implemented using the CONN toolbox (Whitfield-Gabrieli and Nieto-Castanon, 2012), and 49 FCNs at varying edge-densities between 2 and 50% were constructed for each subject. The nodes in the FCNs correspond to the 200 ROIs defined by the Schaefer atlas (Schaefer et al., 2018). The rs-fMRI preprocessing pipeline and FCN construction methodology is identical to our previous work (Elumalai et al., 2022), wherein some of us applied discrete Ricci curvatures to compare FCNs of individuals with ASD and typically developing controls. We remark that our selection of edge-densities between 2 and 50% is to some extent arbitrary. However, there are two reasons why considering very high edge-densities might not be ideal. First, very high edge-densities would transform the network closer to a complete graph, making the calculation of FRC and ORC trivial, and the subsequent group comparisons irrelevant. Second, taking very high edge-densities may result in edges with negative weights in the resulting FCN, as negative ROI-ROI correlations would be included. The presence of negative correlations could affect the reliability and interpretability of the results.

After comparing FRC and ORC across the FCNs of young and elderly groups, we found age-related whole-brain and region-level differences in FCNs. Next, we used meta-analysis decoding to show that the brain regions with age-related differences in FRC and ORC are associated with the cognitive domains of movement, affective processing and somatosensory processing. We found that FRC of some brain regions with age-related differences exhibit significant correlation with behavioral test scores of affective processing. Finally, we showed that FRC and ORC can capture brain regions whose non-invasive stimulation is known to improve motor performance of older adults.

FRC and ORC are fundamentally defined on the edges in a network. Despite being local measures by definition, the average properties of discrete Ricci curvatures have previously been used to characterize the global organization of structural and functional brain networks (Farooq et al., 2020; Elumalai et al., 2022) as well as financial networks (Sandhu et al., 2016; Samal et al., 2021). In this work, we compared the average edge curvatures of the FCNs in the young and elderly groups, and found that both average edge FRC and average edge ORC are significantly higher in the elderly group. Moreover, we found that the global differences in ORC exist across all the network densities considered in this work. Thus, our results suggest that discrete Ricci curvatures are highly sensitive to age-related changes in the global topological organization of resting-state FCNs.

We determined how the age-related differences in curvature are distributed across the 200 brain regions of the Schaefer atlas. We compared node FRC and node ORC across the FCNs of young and elderly individuals. We found 66 brain regions that show age-related differences in FRC and 53 brain regions that show age-related differences in ORC. Notably, FRC and ORC identify 42 brain regions in common. The age-related differences in FRC are mainly concentrated within the somatomotor, dorsal attention, and salience/ventral attention network, whereas the age-related differences in ORC are concentrated within the somatomotor and dorsal attention network. Farooq et al. (2019) have previously applied ORC to compare the structural brain networks of healthy young and healthy old individuals, and reported that brain regions with differences in ORC are present within the default mode network and visual areas. Thus, the application of discrete Ricci curvatures to functional connectivity networks can identify complementary sets of brain regions related to healthy aging, compared to those identified by applying ORC to structural brain networks.

Next, we used Neurosynth meta-analysis decoding based on a large dataset of fMRI studies to determine the cognitive and behavioral relevance of age-related differences in curvature. We found that the brain regions exhibiting age-related differences in FRC are associated with movement, affective processing and somatosensory processing, whereas the brain regions exhibiting age-related differences in ORC are associated with movement. Previous research suggests deficits in motor performance for older adults (Seidler et al., 2010; Cirillo, 2021) such as difficulties in coordination (Seidler et al., 2002), increased variability in movement (Contreras-Vidal et al., 1998) and slowing of movement (Buckles, 1993). Previous research also suggests that healthy aging is associated with changes in affective processing (Mather, 2012). Specifically, elderly individuals are less emotionally reactive to negative situations (Neupert et al., 2007; Mather, 2012) and display higher emotional maturity compared to young individuals. Further, previous literature also suggests a decline in somatosensory functions such as warmth, touch and vibration with age (Edwards and Fillingim, 2001). Notably, Lautenbacher et al. (2017) in their systematic review and meta-analysis study have shown that pain threshold is higher in elderly individuals. Hence, our results suggest that the regions exhibiting differences in discrete Ricci curvatures in healthy aging populations are associated with the cognitive domains and abilities that are typically known to exhibit age-related changes, even in healthy aging. We remark that this is the first study that uses discrete Ricci curvatures to identify the cognitive and behavioral domains associated with age-related changes in brain networks.

After determining the cognitive and behavioral domains identified by discrete Ricci curvatures, we performed a *post-hoc* analysis to test the associations of discrete Ricci curvatures with altered cognition and behavior in healthy aging. We found that FRC of the nodes in the FCNs shows significant correlation with test scores related to affective processing in healthy individuals. Specifically, we found that higher values of FRC are associated with lower levels of chronic stress. These results demonstrate the ability of FRC to identify brain regions that are functionally relevant to affective processing. We remark that the correlations between FRC and the phenotypic test scores reported in our work may be influenced by both individual differences and group-level differences, and it is difficult to ascertain which factor is the main driver of the observed correlations.

Previous studies on neurocognitive aging have provided ample evidence for age-related impairments in motor performance, which may include difficulties in planning, execution and control of movement and deficits in coordination (Krampe et al., 2002; Sawaki et al., 2003; Zimerman and Hummel, 2010). Non-invasive brain stimulation technologies such as tDCS, tACS, and TMS provide an attractive option to modulate brain function and help preserve motor performance in older adults (Summers et al., 2016; Patel et al., 2019; Lee et al., 2021; Pino-Esteban et al., 2021). The Neurosynth meta-analysis decoding performed in the present work demonstrated the ability of both FRC and ORC to identify brain regions that are associated with movement. We performed a manual curation of non-invasive brain stimulation data to determine whether the regions with age-related differences in curvature hold any clinical significance for the treatment of motor declines in healthy elderly individuals. We found that brain regions with age-related differences in curvature overlap with those brain regions whose non-invasive stimulation with tDCS, tACS, or TMS shows evidence for improvement in the motor performance of healthy elderly. Remarkably, within the somatomotor network, both FRC and ORC were able to detect the brain regions that are clinically relevant according to non-invasive stimulation experiments. These results suggest that discrete Ricci curvatures can be used to generate novel hypothesis about target regions for non-invasive brain stimulation experiments.

We highlight a methodological aspect of applying discrete Ricci curvatures to brain networks and place our results in a broader context. In this work, we found similar results for FRC and ORC while determining brain regions related to healthy aging. However, it is important to note that the two notions differ in their definitions as they capture different properties of the classical Ricci curvature, and while both notions are shown to be correlated for many empirical networks (Samal et al., 2018), one may perform better than the other depending on the type of network under consideration. For example, we previously applied FRC and ORC to identify brain regions related to atypical resting state functional connectivity in autism spectrum disorder (Elumalai et al., 2022). We found that FRC is able to identify more regions, provides better interpretability in terms of behavior, and can detect clinically significant regions that are not captured by ORC. Therefore, future works on the application of discrete Ricci curvatures to brain networks may benefit when different notions of curvature are used together, as it may help gather complementary information about the topological organization of brain networks. Notably, in the present study, we found that FRC can capture more brain regions related to healthy aging compared to ORC within the salience/ventral attention network, whereas ORC is able to capture more regions in the limbic network. Further, at the level of cognitive domains, we found that FRC identifies brain regions related to three domains, namely movement, affective processing and somatosensory processing, whereas ORC identifies brain regions related only to movement.

To summarize, we found that geometry-inspired notions of discrete Ricci curvature can be used to characterize age-related changes in brain functional connectivity, both at the whole-brain level as well as at the level of individual brain regions. Further, we showed that the brain regions captured by curvatures hold clinical relevance for non-invasive brain stimulation interventions that are focused toward preserving motor function of older adults. Future studies could expand beyond healthy aging populations by applying discrete Ricci curvatures to brain networks of age-related neurodegenerative disorders such as Alzheimer's disease and Parkinson's disease, and derive insights about the cognitive domains affected in age-related neurodegenerative disorders.

## Data availability statement

Publicly available datasets were analyzed in this study. This data includes raw rs-fMRI and anatomical scans of 228 participants from the MPI-LEMON dataset, and can be found at: http://fcon_1000.projects.nitrc.org/indi/retro/MPI_LEMON.html. Functional connectivity matrices and networks generated in this study, and all original computer programs are available via the GitHub repository: https://github.com/asamallab/Curvature-FCN-Aging.

## Ethics statement

The human participants included in the MPI-LEMON dataset provided written informed consent prior to any data acquisition, including agreement to share their data anonymously. Further, the original MPI-LEMON study was carried out in accordance with the Declaration of Helsinki and the study protocol was approved by the Ethics Committee at the Medical Faculty of the University of Leipzig (Reference number 154/13-ff). The patients/participants provided their written informed consent to participate in this study.

## Author contributions

AS, YY, PE, and NW designed the research. YY and PE performed the computations and generated the figures. YY, PE, NW, JJ, and AS analyzed the data and wrote the paper. AS supervised the project. All authors contributed to the article and approved the submitted version.

## References

[B1] Achard S. Bullmore E. (2007). Efficiency and cost of economical brain functional networks. PLoS Comput. Biol. 3, e17. 10.1371/journal.pcbi.0030017 17274684 PMC1794324

[B2] Achard S. Delon-Martin C. Vértes P. E. Renard F. Schenck M. Schneider F.. (2012). Hubs of brain functional networks are radically reorganized in comatose patients. Proc. Natl. Acad. Sci. U.S.A. 109, 20608–20613. 10.1073/pnas.1208933109 23185007 PMC3528500

[B3] Addis D. R. Barense M. Duarte A. (2015). The Wiley Handbook on the Cognitive Neuroscience of Memory. John Wiley & Sons. 10.1002/9781118332634

[B4] Andersson J. L. Hutton C. Ashburner J. Turner R. Friston K. (2001). Modeling geometric deformations in EPI time series. NeuroImage 13, 903–919. 10.1006/nimg.2001.0746 11304086

[B5] Ashburner J. Friston K. J. (2005). Unified segmentation. NeuroImage 26, 839–851. 10.1016/j.neuroimage.2005.02.018 15955494

[B6] Babayan A. Erbey M. Kumral D. Reinelt J. D. Reiter A. M. Röbbig J.. (2019). A mind-brain-body dataset of MRI, EEG, cognition, emotion, and peripheral physiology in young and old adults. Sci. Data 6, 1–21. 10.1038/sdata.2018.308 30747911 PMC6371893

[B7] Bassett D. S. Nelson B. G. Mueller B. A. Camchong J. Lim K. O. (2012). Altered resting state complexity in schizophrenia. NeuroImage 59, 2196–2207. 10.1016/j.neuroimage.2011.10.002 22008374 PMC3254701

[B8] Bassett D. S. Sporns O. (2017). Network neuroscience. Nat. Neurosci. 20, 353–364. 10.1038/nn.4502 28230844 PMC5485642

[B9] Benjamini Y. Hochberg Y. (1995). Controlling the false discovery rate: a practical and powerful approach to multiple testing. J. R. Stat. Soc. Ser. B 57, 289–300. 10.1111/j.2517-6161.1995.tb02031.x

[B10] Bianconi G. Rahmede C. (2017). Emergent hyperbolic network geometry. Sci. Rep. 7, 1–9. 10.1038/srep41974 28167818 PMC5294422

[B11] Blondel V. D. Guillaume J.-L. Lambiotte R. Lefebvre E. (2008). Fast unfolding of communities in large networks. J. Stat. Mech. 2008, P10008. 10.1088/1742-5468/2008/10/P1000821517554

[B12] Boguñá M. Bonamassa I. De Domenico M. Havlin S. Krioukov D. Ángeles Serrano M. (2021). Network geometry. Nat. Rev. Phys. 3, 114–135. 10.1038/s42254-020-00264-4

[B13] Buckles V. D. (1993). Age-Related Slowing. Dordrecht: Springer Netherlands, 73–87. 10.1007/978-94-011-1976-4_6

[B14] Cabeza R. Nyberg L. Park D. C. (2016). Cognitive Neuroscience of Aging: Linking Cognitive and Cerebral Aging, 2nd Edn. New York, NY: Oxford Academic. 10.1093/acprof:oso/9780199372935.001.0001

[B15] Chai X. J. Casta nón A. N. Öngür D. Whitfield-Gabrieli S. (2012). Anticorrelations in resting state networks without global signal regression. NeuroImage 59, 1420–1428. 10.1016/j.neuroimage.2011.08.048 21889994 PMC3230748

[B16] Chatterjee T. Albert R. Thapliyal S. Azarhooshang N. DasGupta B. (2021). Detecting network anomalies using Forman–Ricci curvature and a case study for human brain networks. Sci. Rep. 11, 8121. 10.1038/s41598-021-87587-z 33854129 PMC8046810

[B17] Cieslik E. C. Zilles K. Caspers S. Roski C. Kellermann T. S.. (2013). Is there “one” DLPFC in cognitive action control? Evidence for heterogeneity from co-activation-based parcellation. Cereb. Cortex 23, 2677–2689. 10.1093/cercor/bhs256 22918987 PMC3792742

[B18] Ciric R. Wolf D. H. Power J. D. Roalf D. R. Baum G. L. Ruparel K.. (2017). Benchmarking of participant-level confound regression strategies for the control of motion artifact in studies of functional connectivity. NeuroImage 154, 174–187. 10.1016/j.neuroimage.2017.03.020 28302591 PMC5483393

[B19] Cirillo J. (2021). Physical activity, motor performance and skill learning: a focus on primary motor cortex in healthy aging. Exp. Brain Res. 239, 3431–3438. 10.1007/s00221-021-06218-1 34499187

[B20] Contreras-Vidal J. L. Teulings H. Stelmach G. (1998). Elderly subjects are impaired in spatial coordination in fine motor control. Acta Psychol. 100, 25–35. 10.1016/S0001-6918(98)00023-7 9844554

[B21] Edwards R. R. Fillingim R. B. (2001). Age-associated differences in responses to noxious stimuli. J. Gerontol. Ser. A Biol. Sci. Med. Sci. 56, M180–M185. 10.1093/gerona/56.3.M180 11253160

[B22] Elumalai P. Yadav Y. Williams N. Saucan E. Jost J. Samal A. (2022). Graph Ricci curvatures reveal atypical functional connectivity in autism spectrum disorder. Sci. Rep. 12, 8295. 10.1038/s41598-022-12171-y 35585156 PMC9117309

[B23] Eyler L. T. Sherzai A. Kaup A. R. Jeste D. V. (2011). A review of functional brain imaging correlates of successful cognitive aging. Biol. Psychiatry 70, 115–122. 10.1016/j.biopsych.2010.12.032 21316037 PMC3641853

[B24] Farooq H. Chen Y. Georgiou T. T. Tannenbaum A. Lenglet C. (2019). Network curvature as a hallmark of brain structural connectivity. Nat. Commun. 10, 4937. 10.1038/s41467-019-12915-x 31666510 PMC6821808

[B25] Farooq H. Lenglet C. Nelson F. (2020). Robustness of brain structural networks is affected in cognitively impaired MS patients. Front. Neurol. 11, 606478. 10.3389/fneur.2020.606478 33329369 PMC7710804

[B26] Forman R. (2003). Bochner's method for cell complexes and combinatorial Ricci curvature. Discrete Comput. Geometry 29, 323–374. 10.1007/s00454-002-0743-x

[B27] Friston K. J. Williams S. Howard R. Frackowiak R. S. J. Turner R. (1996). Movement-related effects in fMRI time-series: movement artifacts in fMRI. Magn. Reson. Med. 35, 346–355. 10.1002/mrm.1910350312 8699946

[B28] Geerligs L. Renken R. J. Saliasi E. Maurits N. M. Lorist M. M. (2015). A brain-wide study of age-related changes in functional connectivity. Cereb. Cortex 25, 1987–1999. 10.1093/cercor/bhu012 24532319

[B29] Grady C. (2012). The cognitive neuroscience of ageing. Nat. Rev. Neurosci. 13, 491–505. 10.1038/nrn3256 22714020 PMC3800175

[B30] Hagberg A. A. Schult D. A. Swart P. J. (2008). “Exploring network structure, dynamics, and function using networkx,” in Proceedings of the 7th Python in Science Conference, eds G. Varoquaux, T. Vaught, and J. Millman (Pasadena, CA; Los Alamos, NM: Los Alamos National Lab), 11–15.

[B31] Harlalka V. Bapi R. S. Vinod P. K. Roy D. (2018). Age, disease, and their interaction effects on intrinsic connectivity of children and adolescents in autism spectrum disorder using functional connectomics. Brain Connect. 8, 407–419. 10.1089/brain.2018.0616 30009617

[B32] Itahashi T. Yamada T. Watanabe H. Nakamura M. Jimbo D. Shioda S.. (2014). Altered network topologies and hub organization in adults with autism: a resting-state fMRI study. PLoS ONE 9, e94115. 10.1371/journal.pone.0094115 24714805 PMC3979738

[B33] Jost J. (2017). Riemannian Geometry and Geometric Analysis, 7th Edn. New York, NY: Springer Berlin Heidelberg. 10.1007/978-3-319-61860-9

[B34] Kartun-Giles A. P. Bianconi G. (2019). Beyond the clustering coefficient: a topological analysis of node neighbourhoods in complex networks. Chaos Solitons Fract. 1, 100004. 10.1016/j.csfx.2019.100004

[B35] Krampe R. T. Engbert R. Kliegl R. (2002). The effects of expertise and age on rhythm production: adaptations to timing and sequencing constraints. Brain Cogn. 48, 179–194. 10.1006/brcg.2001.1312 11812041

[B36] Kruskal J. B. (1956). On the shortest spanning subtree of a graph and the traveling salesman problem. Proc. Am. Math. Soc. 7, 48. 10.1090/S0002-9939-1956-0078686-730656504

[B37] Latora V. Marchiori M. (2001). Efficient behavior of small-world networks. Phys. Rev. Lett. 87, 198701. 10.1103/PhysRevLett.87.198701 11690461

[B38] Lautenbacher S. Peters J. H. Heesen M. Scheel J. Kunz M. (2017). Age changes in pain perception: a systematic-review and meta-analysis of age effects on pain and tolerance thresholds. Neurosci. Biobehav. Rev. 75, 104–113. 10.1016/j.neubiorev.2017.01.039 28159611

[B39] Lee J. H. Lee T. L. Kang N. (2021). Transcranial direct current stimulation decreased cognition-related reaction time in older adults: a systematic review and meta-analysis. Ageing Res. Rev. 70, 101377. 10.1016/j.arr.2021.101377 34089900

[B40] Li W. Wang M. Li Y. Huang Y. Chen X. (2016). A novel brain network construction method for exploring age-related functional reorganization. Comput. Intell. Neurosci. 2016, 2429691. 10.1155/2016/2429691 27057155 PMC4789383

[B41] Lin Y. Lu L. Yau S.-T. (2011). Ricci curvature of graphs. Tohoku Math. J. 63, 605–627. 10.2748/tmj/1325886283

[B42] Logothetis N. K. (2008). What we can do and what we cannot do with fMRI. Nature 453, 869–878. 10.1038/nature06976 18548064

[B43] Lohmann G. Lacosse E. Ethofer T. Kumar V. J. Scheffler K. Jost J. (2021). Predicting intelligence from fmri data of the human brain in a few minutes of scan time. bioRxiv. 10.1101/2021.03.18.435935 [preprint].

[B44] Luppi A. I. Stamatakis E. A. (2021). Combining network topology and information theory to construct representative brain networks. Netw. Neurosci. 5, 96–124. 10.1162/netn_a_00170 33688608 PMC7935031

[B45] Mather M. (2012). The emotion paradox in the aging brain. Ann. N. Y. Acad. Sci. 1251, 33–49. 10.1111/j.1749-6632.2012.06471.x 22409159 PMC3395773

[B46] Moher D. Liberati A. Tetzlaff J. Altman D. G. Group P. (2009). Preferred reporting items for systematic reviews and meta-analyses: the prisma statement. PLoS Med. 6, e1000097. 10.1371/journal.pmed.1000097 21603045 PMC3090117

[B47] Neupert S. D. Almeida D. M. Charles S. T. (2007). Age differences in reactivity to daily stressors: the role of personal control. J. Gerontol. Ser. B Psychol. Sci. Soc. Sci. 62, P216–P225. 10.1093/geronb/62.4.P216 17673531

[B48] Ni C. Lin Y. Gao J. Gu X. D. Saucan E. (2015). “Ricci curvature of the Internet topology,” in 2015 IEEE Conference on Computer Communications (INFOCOM) (Hong Kong), 2758–2766. 10.1109/INFOCOM.2015.7218668

[B49] Ni C.-C. Lin Y.-Y. Luo F. Gao J. (2019). Community detection on networks with Ricci flow. Sci. Rep. 9, 9984. 10.1038/s41598-019-46380-9 31477786 PMC6718387

[B50] Nitsche M. A. Cohen L. G. Wassermann E. M. Priori A. Lang N. Antal A.. (2008). Transcranial direct current stimulation: state of the art 2008. Brain Stimul. 1, 206–223. 10.1016/j.brs.2008.06.004 20633386

[B51] Nyberg L. Salami A. Andersson M. Eriksson J. Kalpouzos G. Kauppi K.. (2010). Longitudinal evidence for diminished frontal cortex function in aging. Proc. Natl. Acad. Sci. U.S.A. 107, 22682–22686. 10.1073/pnas.1012651108 21156826 PMC3012514

[B52] Ollivier Y. (2007). Ricci curvature of metric spaces. Comptes Rendus Math. 345, 643–646. 10.1016/j.crma.2007.10.041

[B53] Patel R. Ashcroft J. Patel A. Ashrafian H. Woods A. J. Singh H.. (2019). The impact of transcranial direct current stimulation on upper-limb motor performance in healthy adults: a systematic review and meta-analysis. Front. Neurosci. 13, 1213. 10.3389/fnins.2019.01213 31803003 PMC6873898

[B54] Petrowski K. Braehler E. Schmalbach B. Hinz A. Bastianon C. Ritz T. (2020). Psychometric properties of an english short version of the trier inventory for chronic stress. BMC Med. Res. Methodol. 20, 1–8. 10.1186/s12874-020-01156-y 33323110 PMC7739474

[B55] Pino-Esteban A. Megía-García Á. Martín-Caro Álvarez D. Beltran-Alacreu H. Avendaño-Coy J. Gómez-Soriano J.. (2021). Can transcranial direct current stimulation enhance functionality in older adults? A systematic review. J. Clin. Med. 10, 2981. 10.3390/jcm10132981 34279465 PMC8268136

[B56] Porcu M. Cocco L. Puig J. Mannelli L. Yang Q. Suri J. S.. (2021). Global fractional anisotropy: effect on resting-state neural activity and brain networking in healthy participants. Neuroscience 472, 103–115. 10.1016/j.neuroscience.2021.07.021 34364954

[B57] Porcu M. Operamolla A. Scapin E. Garofalo P. Destro F. Caneglias A.. (2020). Effects of white matter hyperintensities on brain connectivity and hippocampal volume in healthy subjects according to their localization. Brain Connect. 10, 436–447. 10.1089/brain.2020.0774 32842755

[B58] Power J. D. Mitra A. Laumann T. O. Snyder A. Z. Schlaggar B. L. Petersen S. E. (2014). Methods to detect, characterize, and remove motion artifact in resting state fMRI. NeuroImage 84, 320–341. 10.1016/j.neuroimage.2013.08.048 23994314 PMC3849338

[B59] Reis J. Schambra H. M. Cohen L. G. Buch E. R. Fritsch B. Zarahn E.. (2009). Noninvasive cortical stimulation enhances motor skill acquisition over multiple days through an effect on consolidation. Proc. Natl. Acad. Sci. U.S.A. 106, 1590–1595. 10.1073/pnas.0805413106 19164589 PMC2635787

[B60] Ritchie H. Roser M. (2019). Age structure. Our World in Data. Available online at: https://ourworldindata.org/age-structure

[B61] Rodriguez-Sabate C. Morales I. Rodriguez M. (2022). The influence of aging on the functional connectivity of the human basal ganglia. Front. Aging Neurosci. 2021, 785666. 10.3389/fnagi.2021.785666 35095470 PMC8789673

[B62] Rorden C. Karnath H.-O. Bonilha L. (2007). Improving lesion-symptom mapping. J. Cogn. Neurosci. 19, 1081–1088. 10.1162/jocn.2007.19.7.1081 17583985

[B63] Rudie J. D. Brown J. Beck-Pancer D. Hernandez L. Dennis E. Thompson P.. (2013). Altered functional and structural brain network organization in autism. NeuroImage 2, 79–94. 10.1016/j.nicl.2012.11.006 24179761 PMC3777708

[B64] Sala-Llonch R. Junqué C. Arenaza-Urquijo E. M. Vidal-Piñeiro D. Valls-Pedret C. Palacios E. M.. (2014). Changes in whole-brain functional networks and memory performance in aging. Neurobiol. Aging 35, 2193–2202. 10.1016/j.neurobiolaging.2014.04.007 24814675

[B65] Samal A. Pharasi H. K. Ramaia S. J. Kannan H. Saucan E. (2021). Network geometry and market instability. R. Soc. Open Sci. 8, 201734. 10.1098/rsos.201734 33972862 PMC8074692

[B66] Samal A. Sreejith R. Gu J. Liu S. Saucan E. Jost J. (2018). Comparative analysis of two discretizations of Ricci curvature for complex networks. Sci. Rep. 8, 8650. 10.1038/s41598-018-27001-3 29872167 PMC5988801

[B67] Sandhu R. S. Georgiou T. T. Tannenbaum A. R. (2016). Ricci curvature: an economic indicator for market fragility and systemic risk. Sci. Adv. 2, e1501495. 10.1126/sciadv.1501495 27386522 PMC4928924

[B68] Sawaki L. Yaseen Z. Kopylev L. Cohen L. G. (2003). Age-dependent changes in the ability to encode a novel elementary motor memory. Ann. Neurol. 53, 521–524. 10.1002/ana.10529 12666120

[B69] Schaefer A. Kong R. Gordon E. M. Laumann T. O. Zuo X.-N. Holmes A. J.. (2018). Local-global parcellation of the human cerebral cortex from intrinsic functional connectivity MRI. Cereb. Cortex 28, 3095–3114. 10.1093/cercor/bhx179 28981612 PMC6095216

[B70] Schulz P. Schlotz W. Becker P. (2004). Trierer Inventar zum Chronischen Stress (TICS). Göttingen: Hogrefe.

[B71] Seabold S. Perktold J. (2010). “statsmodels: Econometric and statistical modeling with python,” in 9th Python in Science Conference (Austin, TX). 10.25080/Majora-92bf1922-011

[B72] Seidler R. D. Alberts J. L. Stelmach G. E. (2002). Changes in multi-joint performance with age. Motor Control 6, 19–31. 10.1123/mcj.6.1.19 11842268

[B73] Seidler R. D. Bernard J. A. Burutolu T. B. Fling B. W. Gordon M. T. Gwin J. T.. (2010). Motor control and aging: links to age-related brain structural, functional, and biochemical effects. Neurosci. Biobehav. Rev. 34, 721–733. 10.1016/j.neubiorev.2009.10.005 19850077 PMC2838968

[B74] Sia J. Jonckheere E. Bogdan P. (2019). Ollivier-ricci curvature-based method to community detection in complex networks. Sci. Rep. 9, 9800. 10.1038/s41598-019-46079-x 31278341 PMC6611887

[B75] Simhal A. K. Carpenter K. L. Kurtzberg J. Song A. Tannenbaum A. Zhang L.. (2022). Changes in the geometry and robustness of diffusion tensor imaging networks: secondary analysis from a randomized controlled trial of young autistic children receiving an umbilical cord blood infusion. Front. Psychiatry 13, 1026279. 10.3389/fpsyt.2022.1026279 36353577 PMC9637553

[B76] Simhal A. K. Carpenter K. L. Nadeem S. Kurtzberg J. Song A. Tannenbaum A.. (2020). Measuring robustness of brain networks in autism spectrum disorder with Ricci curvature. Sci. Rep. 10, 10819. 10.1038/s41598-020-67474-9 32616759 PMC7331646

[B77] Sladky R. Friston K. J. Tröstl J. Cunnington R. Moser E. Windischberger C. (2011). Slice-timing effects and their correction in functional MRI. NeuroImage 58, 588–594. 10.1016/j.neuroimage.2011.06.078 21757015 PMC3167249

[B78] Song J. Birn R. M. Boly M. Meier T. B. Nair V. A. Meyerand M. E.. (2014). Age-related reorganizational changes in modularity and functional connectivity of human brain networks. Brain Connect. 4, 662–676. 10.1089/brain.2014.0286 25183440 PMC4238253

[B79] Spreng R. N. Wojtowicz M. Grady C. L. (2010). Reliable differences in brain activity between young and old adults: a quantitative meta-analysis across multiple cognitive domains. Neurosci. Biobehav. Rev. 34, 1178–1194. 10.1016/j.neubiorev.2010.01.009 20109489

[B80] Sreejith R. P. Mohanraj K. Jost J. Saucan E. Samal A. (2016). Forman curvature for complex networks. J. Stat. Mech. 2016, 063206. 10.1088/1742-5468/2016/06/063206

[B81] Strotzer M. (2009). One century of brain mapping using brodmann areas. Clin. Neuroradiol. 19, 179–186. 10.1007/s00062-009-9002-3 19727583

[B82] Summers J. J. Kang N. Cauraugh J. H. (2016). Does transcranial direct current stimulation enhance cognitive and motor functions in the ageing brain? A systematic review and meta-analysis. Ageing Res. Rev. 25, 42–54. 10.1016/j.arr.2015.11.004 26607412

[B83] Tian Y. Lubberts Z. Weber M. (2022). “Mixed-membership community detection via line graph curvature,” in NeurIPS 2022 Workshop on Symmetry and Geometry in Neural Representations (New Orleans). Available online at: https://www.neurreps.org/

[B84] Tromp D. Dufour A. Lithfous S. Pebayle T. Després O. (2015). Episodic memory in normal aging and alzheimer disease: insights from imaging and behavioral studies. Ageing Res. Rev. 24, 232–262. 10.1016/j.arr.2015.08.006 26318058

[B85] United Nations Department of Economic and Social Affairs Population Division. (2022). World Population Prospects 2022: Summary of Results. UN DESA/POP/2022/TR/NO. 3.

[B86] Vaserstein L. N. (1969). Markov processes over denumerable products of spaces, describing large systems of automata. Probl. Peredachi Inf. 5, 64–72.

[B87] Virtanen P. Gommers R. Oliphant T. E. Haberland M. Reddy T. Cournapeau D.. (2020). SciPy 1.0: fundamental algorithms for scientific computing in Python. Nat. Methods 17, 261–272. 10.1038/s41592-019-0686-2 32015543 PMC7056644

[B88] Whitfield-Gabrieli S. Nieto-Castanon A. (2012). Conn : a functional connectivity toolbox for correlated and anticorrelated brain networks. Brain Connect. 2, 125–141. 10.1089/brain.2012.0073 22642651

[B89] Whitlock J. R. (2017). Posterior parietal cortex. Curr. Biol. 27, R691–R695. 10.1016/j.cub.2017.06.007 28743011

[B90] Williams N. Wang S. Arnulfo G. Nobili L. Palva S. Palva J. (2021). Modules in connectomes of phase-synchronization comprise anatomically contiguous, functionally related regions. bioRxiv. 10.1101/2021.06.24.449415 [preprint]. 36966852

[B91] Xia M. Wang J. He Y. (2013). Brainnet viewer: a network visualization tool for human brain connectomics. PLoS ONE 8, e68910. 10.1371/journal.pone.0068910 23861951 PMC3701683

[B92] Xu T. Cullen K. R. Mueller B. Schreiner M. W. Lim K. O. Schulz S. C.. (2016). Network analysis of functional brain connectivity in borderline personality disorder using resting-state fMRI. NeuroImage 11, 302–315. 10.1016/j.nicl.2016.02.006 26977400 PMC4782004

[B93] Yarkoni T. Poldrack R. A. Nichols T. E. Van Essen D. C. Wager T. D. (2011). Large-scale automated synthesis of human functional neuroimaging data. Nat. Methods 8, 665–670. 10.1038/nmeth.1635 21706013 PMC3146590

[B94] Zimerman M. Hummel F. C. (2010). Non-invasive brain stimulation: enhancing motor and cognitive functions in healthy old subjects. Front. Aging Neurosci. 2, 149. 10.3389/fnagi.2010.00149 21151809 PMC2999819

