# Supplementary material for: Discrete Ricci curvatures capture age-related changes in human brain functional connectivity networks
Source: Front Aging Neurosci. 2023 May 24;15:1120846. doi: 10.3389/fnagi.2023.1120846 (PMC10244515; doi:10.3389/fnagi.2023.1120846)
Supplement: Supplementary file 2 [file Data_Sheet_1.pdf]

SUPPLEMENTARY MATERIAL
for
Discrete Ricci curvatures capture age-related changes in human brain functional connectivity networks

Yasharth Yadav [1,2], Pavithra Elumalai [1], Nitin Williams [3,4], Jürgen Jost [5,6], and Areejit Samal [1,7,*]

[1] *The Institute of Mathematical Sciences (IMSc), Chennai, India*
[2] *Indian Institute of Science Education and Research (IISER), Pune, India*
[3] *Helsinki Institute of Information Technology, Department of Computer Science, Aalto University, Finland*
[4] *Department of Neuroscience & Biomedical Engineering, Aalto University, Finland*
[5] *Max Planck Institute for Mathematics in the Sciences, Leipzig, Germany*
[6] *The Santa Fe Institute, Santa Fe, NM, USA*
[7] *Homi Bhabha National Institute (HBNI), Mumbai, India*

Correspondence*:
Areejit Samal
asamal@imsc.res.in

STANDARD NETWORK MEASURES

All the standard network measures are defined for an unweighted and undirected graph $G = (V, E)$. The graph G can also be denoted as $n \times n$ adjacency matrix $\mathbf{A}$, where $A_{ij} = 1$ if nodes i and j are adjacent, and $A_{ij} = 0$ otherwise.

- The *clique number* of G is defined as the size of the maximal clique appearing in G.

- For a node i in the graph G, *clustering coefficient* is defined as:

$$C_i = \frac{2}{k_i(k_i - 1)} \sum_{j,k} (A_{ij} A_{ik} A_{jk})^{1/3}$$

  where j and k are the neighbors of node i and the summation is over all pairs of neighbors of i. The *average clustering coefficent* of G is the average of the clustering coefficients of the individual nodes in G.

- The *shortest path length* $d(i, j)$ between any two nodes i and j in G is equal to the number of edges contained in the shortest path connecting them. *Average shortest path length* of G is the average of the

shortest path length between all pairs of nodes in G, i.e.,

$$\langle L \rangle = \frac{1}{n(n-1)} \sum_{i,j \in V} d(i,j)$$

- *Global efficiency* measures the ability of the network G to exchange information (Latora and Marchiori, 2001), and is defined as

$$E_{glob}(G) = \frac{1}{n(n-1)} \sum_{i \neq j \in V} \frac{1}{d(i,j)}.$$

- Let G_i denote the subgraph of the neighbors of node i in G. The *local efficiency* (Latora and Marchiori, 2001) of node i, $E_{loc}(G_i)$ is defined as the efficiency of the subgraph G_i. *Average local efficiency* of G is given by

$$E_{loc}(G) = \frac{1}{n} \sum_{i \in V} E_{loc}(G_i)$$

- The *betweenness centrality* of a node i in G measures the extent to which it lies on the shortest path between other nodes, and is defined as (Freeman, 1977)

$$C_b(i) = \sum_{j,k \in V} \frac{\sigma(j,k|i)}{\sigma(j,k)},$$

  where $\sigma(j,k)$ is the number of shortest paths between j and k, and $\sigma(j,k|i)$ is the number of shortest paths between j and k that pass through i.

- Many networks display a tendency to exhibit a modular structure, where the set of nodes in a network can be partitioned into subsets of densely connected nodes. *Modularity* of the graph G measures the density of intra-module edges compared to inter-module edges, and is defined as (Girvan and Newman, 2002; Blondel et al., 2008)

$$Q = \frac{1}{2m_w} \sum_{i \neq j \in V} [A_{ij} - \frac{s_i s_j}{2m_w}] \delta(c_i, c_j)$$

  where s_i and s_j give the sum of weights of edges attached to nodes i and j, respectively, c_i and c_j are the communities of i and j, respectively, and m_w is the sum of all edge weights. Since G is an unweighted graph, all edges in G are assigned weight equal to 1.

- A network is said to be assortative when two nodes attached to a given edge in the network tend to have the same degree. The *assortativity coefficient* (Newman, 2003) measures the degree correlations

between nodes in an unweighted network G, and is defined as

$$r = \frac{\sum_i e_{ii} - \sum_i a_i b_i}{1 - \sum_i a_i b_i}.$$

Here, e_{ij} is the fraction of edges in G that are attached to nodes of degree i and j. Note that $\sum_{ij} e_{ij} = 1$. Further, a_i and b_i are the fraction of edges whose one end is attached to nodes of degree i, that is $a_i = \sum_j e_{ij}$ and $b_i = \sum_j e_{ji}$. Since G is an undirected network, we have $e_{ij} = e_{ji}$ and $a_i = b_i$.

REFERENCES

Blondel, V. D., Guillaume, J.-L., Lambiotte, R., and Lefebvre, E. (2008). Fast unfolding of communities in large networks. *Journal of Statistical Mechanics: Theory and Experiment* 2008, P10008. doi:10.1088/1742-5468/2008/10/P10008

Freeman, L. C. (1977). A Set of Measures of Centrality Based on Betweenness. *Sociometry* 40, 35. doi:10.2307/3033543

Girvan, M. and Newman, M. E. J. (2002). Community structure in social and biological networks. *Proceedings of the National Academy of Sciences* 99, 7821–7826. doi:10.1073/pnas.122653799

Latora, V. and Marchiori, M. (2001). Efficient Behavior of Small-World Networks. *Physical Review Letters* 87, 198701. doi:10.1103/PhysRevLett.87.198701

Moher, D., Liberati, A., Tetzlaff, J., Altman, D. G., and Group, P. (2009). Preferred reporting items for systematic reviews and meta-analyses: the prisma statement. *PLoS Medicine* 6, e1000097. doi:10.1371/journal.pmed.1000097

Newman, M. E. (2003). Mixing patterns in networks. *Physical Review E* 67, 026126. doi:10.1103/PhysRevE.67.026126

SUPPLEMENTARY FIGURES

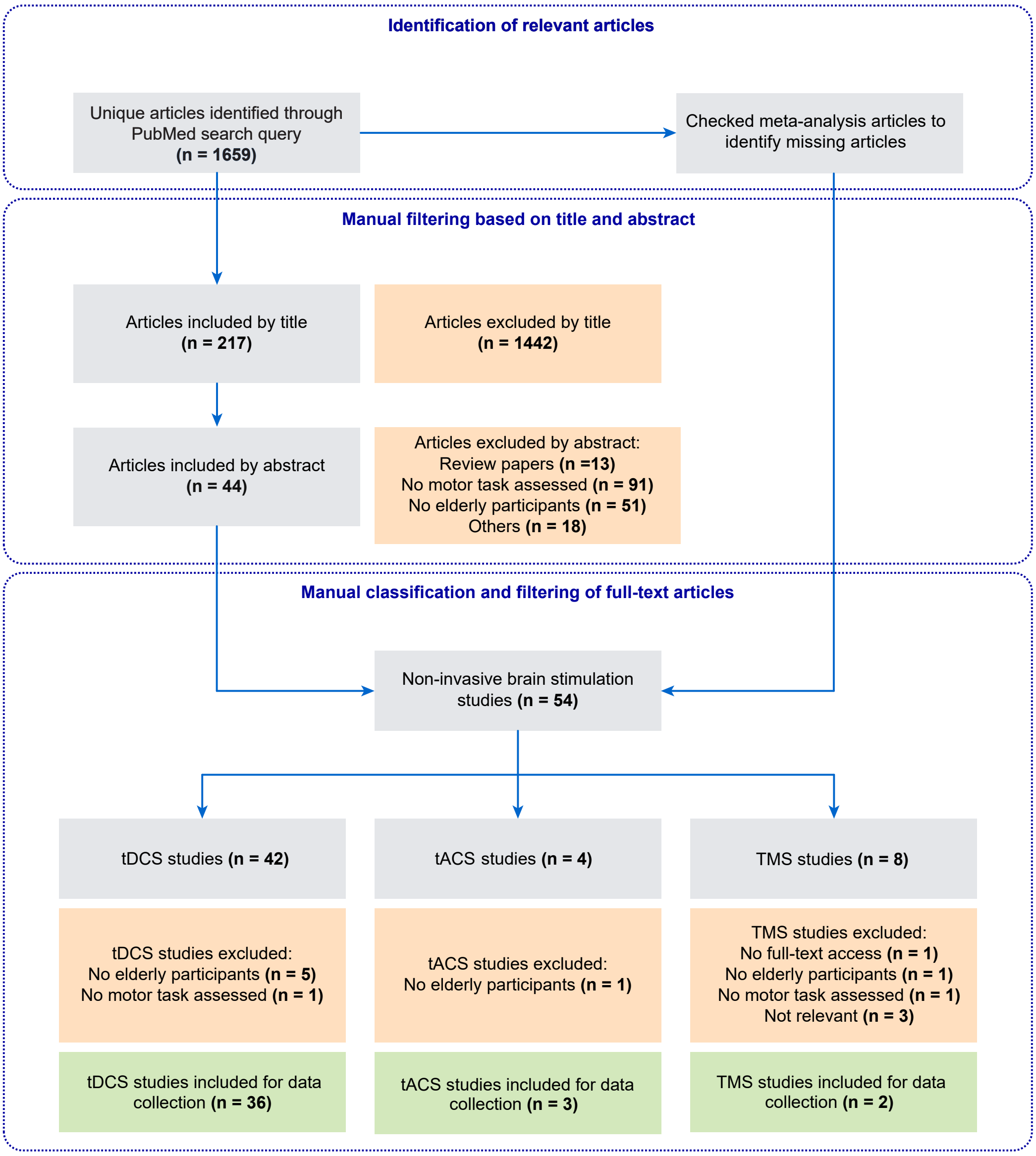

Supplementary Figure 1. Summary of the workflow used to collect and classify data from non-invasive brain stimulation experiments. The workflow is presented according to PRISMA statement (Moher et al., 2009). We obtained a list of 1659 articles from PubMed that report the effects of non-invasive brain stimulation on motor performance during healthy aging. We followed a three-stage procedure to extract relevant articles from the PubMed search. First, we identified missing articles from the original PubMed search by checking meta-analysis papers studying the effects of non-invasive stimulation on motor performance during healthy aging. Second, we filtered the articles based on title and abstract. Third, we classified the articles according to the non-invasive stimulation technique (tDCS/tACS/TMS) and checked the full-text of these articles for relevant data. Finally, we extracted experimental data from 36 tDCS studies, 3 tACS studies and 2 TMS studies.

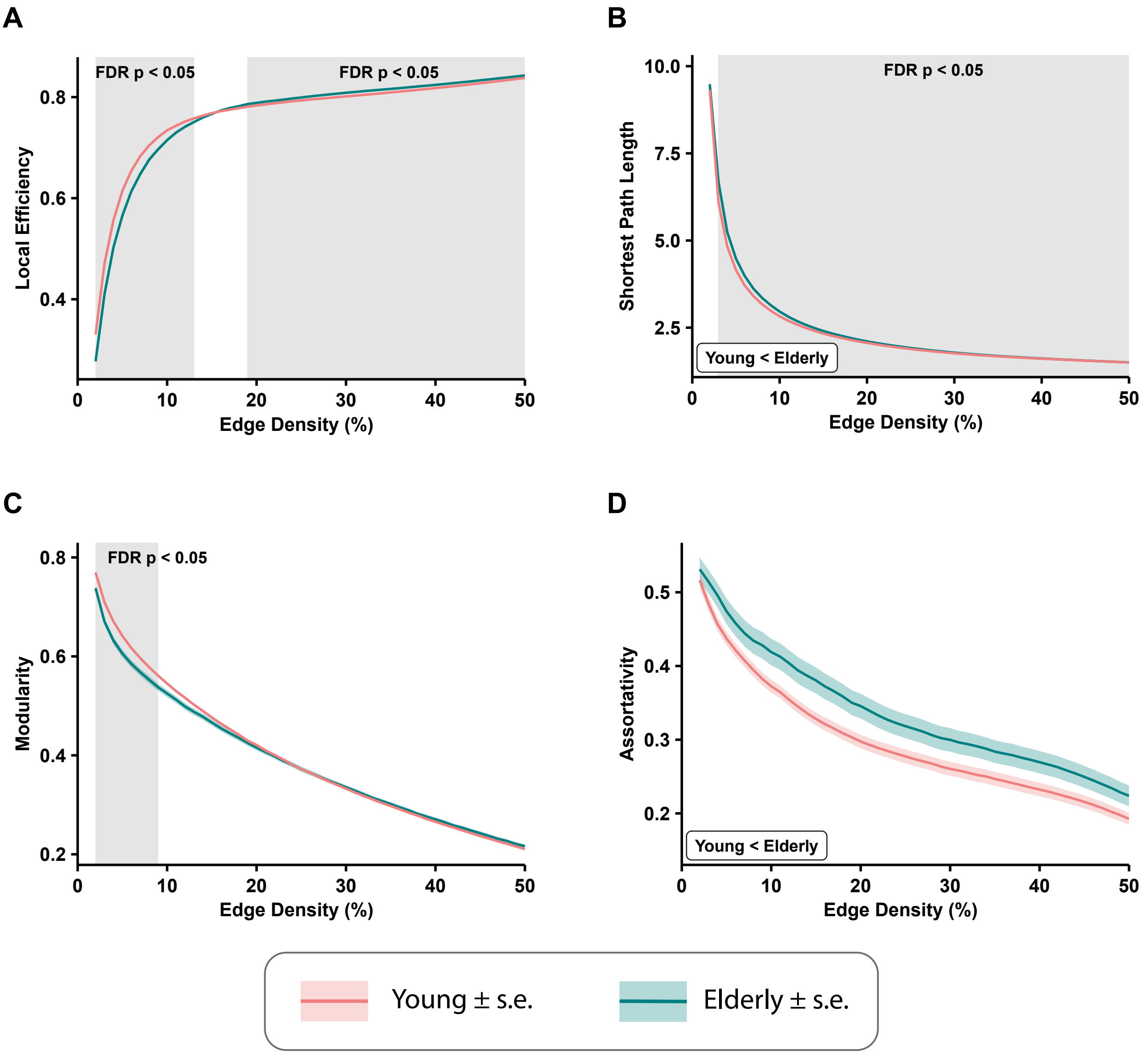

Supplementary Figure 2. Differences in standard global network measures across the functional connectivity networks (FCNs) of 153 young individuals and 72 elderly individuals. The differences are reported for FCNs over the range of edge densities 0.02 (i.e., 2% edges) and 0.5 (i.e., 50% edges), with an increment of 0.01 (i.e., 1% edges). The shaded regions in each plot correspond to the edge densities where the between-group differences are statistically significant ($p < 0.05$, FDR-corrected). **(A)** Average local efficiency is significantly higher in young individuals over edge densities $1 - 12\%$, and significantly higher in elderly individuals over edge densities $19 - 49\%$. **(B)** Average shortest path length is significantly higher in elderly individuals over edge densities $3 - 49\%$. **(C)** Modularity is significantly higher in young individuals over edge densities $2 - 9\%$. **(D)** Assortativity is higher in the young group, but the differences are not statistically significant.

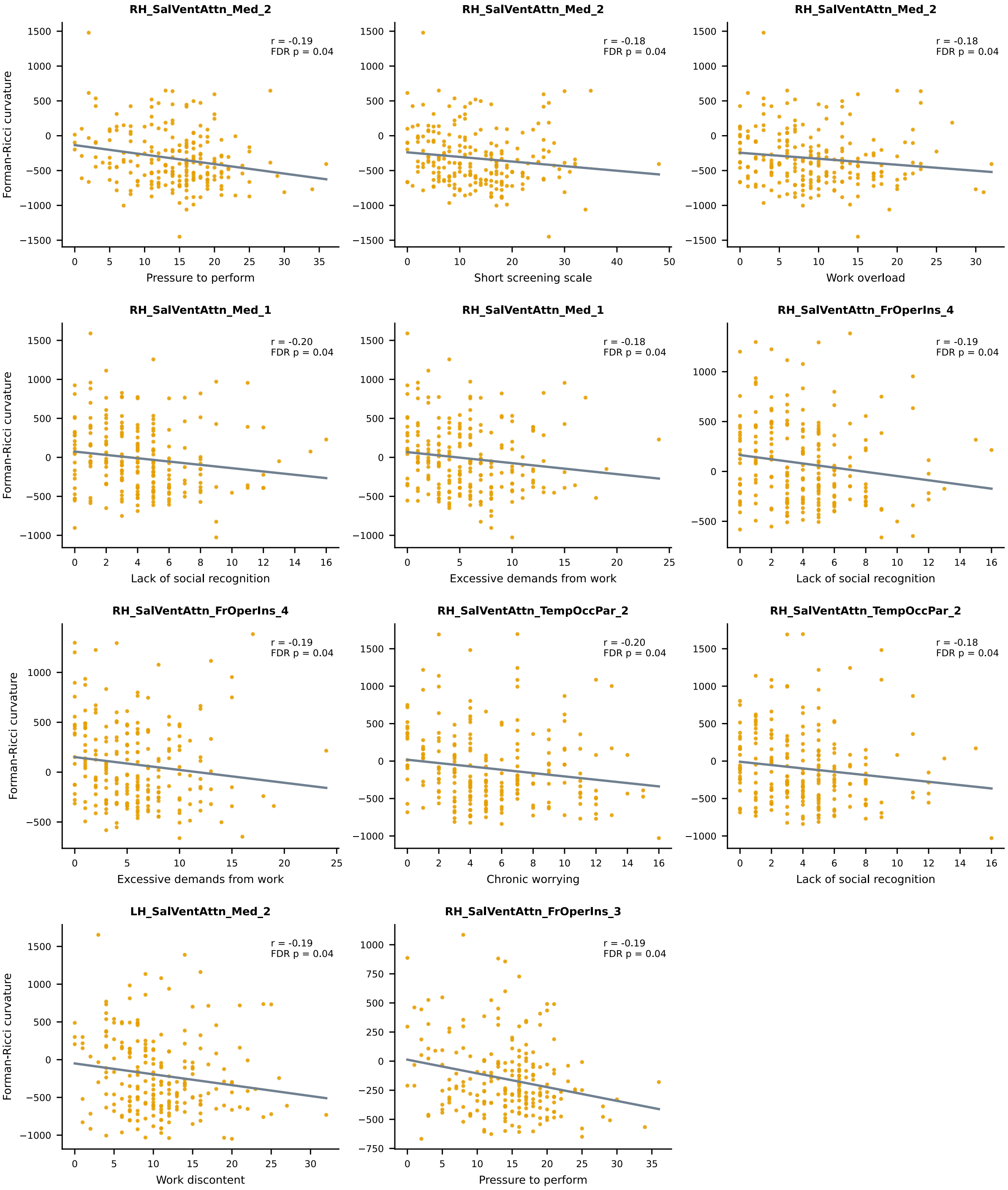

Supplementary Figure 3. Correlation between Forman-Ricci curvature (FRC) and TICS scores of chronic stress. Scatter plots depicting the relationship between FRC of 6 brain regions (RH_SalVentAttn_Med_2, RH_SalVentAttn_Med_1, RH_SalVentAttn_FrOperIns_4, RH_SalVentAttn_TempOccPar_2, LH_SalVentAttn_Med_2 and LH_SalVentAttn_FrOperIns_3), and TICS scores of chronic stress. Note that only the scatter plots corresponding to significant correlations r between FRC and TICS scores ($p < 0.05$, FDR corrected) are shown in this figure. Each plot displays a line describing the linear relationship between FRC of a brain region and the corresponding TICS score, estimated using the least squares method. Brain regions are named according to the labeling scheme provided in the Schaefer atlas.
